# Investigating Molecular Adsorption on Graphene-Supported Platinum Subnanoclusters: Insights from DFT + D3 Calculations

**DOI:** 10.1021/acsomega.4c07017

**Published:** 2024-09-18

**Authors:** João Paulo Cerqueira Felix, Gabriel Reynald da Silva, Glaucio R. Nagurniak, Alexandre C Dias, Renato P Orenha, Celso R. C. Rêgo, Renato L. T. Parreira, Diego Guedes-Sobrinho, Maurício J. Piotrowski

**Affiliations:** † Institute of Physics “Armando Dias Tavares”, Rio de Janeiro State University, 20550-900 Rio de Janeiro, RJ, Brazil; ‡ Chemistry Department, Federal University of Paraná, 81531-980 Curitiba, PR, Brazil; § Department of Exact Sciences and Education, Federal University of Santa Catarina, 89036-004 Blumenau, SC, Brazil; ∥ Institute of Physics and International Center of Physics, University of Brasília, 70919-970 Brasília, DF, Brazil; ⊥ Núcleo de Pesquisas em Ciências Exatas e Tecnológicas, Universidade de Franca, 14404-600 Franca, SP, Brazil; # Institute of Nanotechnology Hermann-von-Helmholtz-Platz, Karlsruhe Institute of Technology, 76021 Karlsruhe, Germany; ∇ Department of Physics, Federal University of Pelotas, PO Box 354, 96010-900 Pelotas, RS, Brazil

## Abstract

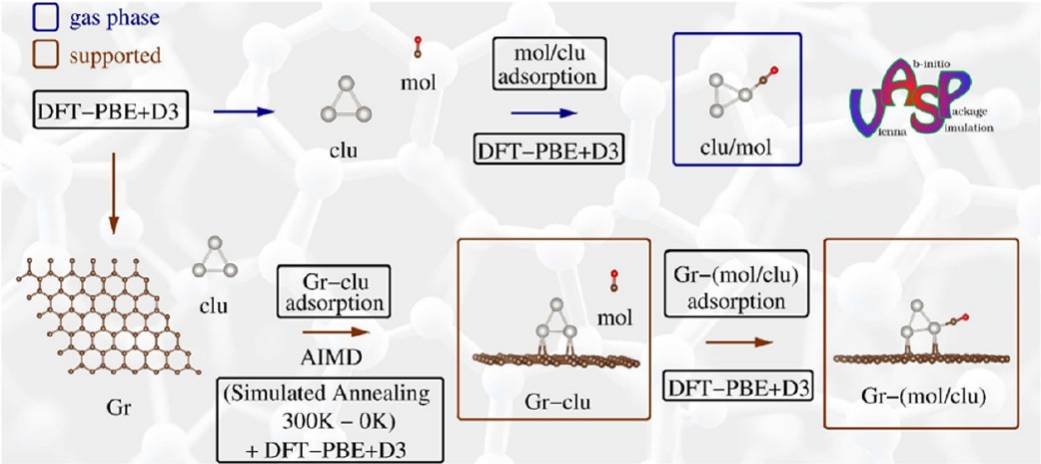

Platinum (Pt) subnanoclusters have become pivotal in nanocatalysis, yet their molecular adsorption mechanisms, particularly on supported versus unsupported systems, remain poorly understood. Our study employs detailed density functional theory (DFT) calculations with D3 corrections to investigate molecular adsorption on Pt subnanoclusters, focusing on CO, NO, N_2_, and O_2_ species. Gas-phase and graphene-supported scenarios are systematically characterized to elucidate adsorption mechanisms and catalytic potential. Gas-phase Pt_*n*_ clusters are first analyzed to identify stable configurations and assess size-dependent reactivity. Transitioning to graphene-supported Pt_*n*_ clusters, both periodic and nonperiodic models are employed to study interactions with graphene substrates. Strong adsorbate interactions predominantly occur at single top sites, revealing distinct adsorption geometries and stabilization effects for specific molecules on Pt_6_ clusters. Energy decomposition analysis highlights the paramount role of graphene substrates in enhancing stability and modulating cluster-adsorbate interactions. The interaction energy emerges as a critical criterion within the Sabatier principle, crucial for distinguishing between physisorption and chemisorption. Hybridization indices and charge density flow tendencies establish direct relationships with stabilization processes, underscoring graphene’s influence in stabilizing highly reactive subnanoclusters. This comprehensive investigation provides critical insights into molecular adsorption mechanisms and the catalytic potential of graphene-supported Pt nanoclusters. Our findings contribute to a deeper understanding of nanocatalysis, emphasizing the essential role of substrates in optimizing catalytic performance for industrial applications.

## 1 Introduction

Metal subnanometric clusters exhibit distinctive physicochemical properties compared to their bulk counterparts.^1,2^ These properties include enhanced reactivity, catalytic efficiency, and unique optical, electronic, structural, and magnetic behaviors, making them highly sought-after in various applications.^3,4^ Among transition metals, particularly 3d, 4d, and 5d metal clusters, platinum (Pt) nanoclusters have garnered significant attention for their catalytic potential.^5^ This is especially evident in processes crucial to pharmaceutical synthesis and chemical manufacturing.^6,7^ Pt nanoclusters are promising eco-friendly nanomaterials at the forefront of contemporary scientific and technological advancement.^8,9^ These materials, studied and manipulated at scales below 1 nm, represent a critical frontier in the quest for sustainable and efficient materials that balance economic viability with technological functionality.^9^

Despite their effectiveness, Pt-based catalysts are notably expensive, limiting their widespread adoption. Understanding the precise physicochemical properties and behaviors of Pt clusters at the nanoscale is essential to mitigate these costs and enhance their efficiency. This can be achieved by synthesizing atomically precise metal clusters, which generate systems with low-coordination unsaturated active surface sites.^4,10,11^ The method of atomic-level precision preparation is crucial, as even a single atom can alter the nanocluster’s reactivity. For example, Imaoka et al.^11^ performed atom-precise and scalable Pt nanocluster synthesis, achieving excellent catalytic performance with the Pt_10_ nanocluster in styrene hydrogenation. Thus, more experimental and theoretical studies are needed to explore the atomic-level comprehension of these subnanometric systems, particularly considering the molecular interaction mechanisms and supporting aspects, as finite-size effects are crucial for the electronic structure of these ultrasmall clusters.

Computational simulations, mainly using density functional theory (DFT) methods, offer a precise means to explore these properties without the constraints of experimental limitations inherent in gas-phase or vacuum methods, which often lead to particle aggregation and reduced control over interactions.^12^ In experimental settings, supporting Pt nanoclusters on substrates such as graphene has emerged as a promising approach.^13−15^ Graphene, a single layer of carbon atoms arranged in a hexagonal honeycomb lattice, possesses exceptional mechanical, thermal, and electronic properties.^16−18^ Being an excellent candidate for a stabilizing substrate, whether pristine or modified, graphene has been extensively studied for the adsorption of transition metal nanoclusters.^19^ Its high electron mobility and robust structure make it an ideal substrate for stabilizing and enhancing the catalytic properties of Pt nanoclusters.^20^

Despite significant research progress,^21−23^ uncertainties persist in understanding the behavior of transition metal subnanoclusters, particularly Pt nanoclusters, interact with molecular species like CO, NO, N_2_, and O_2_ when supported on graphene.^24−27^ Such interactions are crucial as they dictate the catalytic efficiency and stability of these systems in chemical environments.^28,29^ Fundamentally, the interaction mechanisms between molecular species and metallic surfaces are well-comprehended, characterized by donation and back-donation processes.^30−32^ However, a theoretical framework to understand adsorption phenomena for subnanoclusters in different chemical environments demands an additional effort, particularly for Pt nanoclusters (Pt: d^9^s^1^) with their enhanced chemical reactivity, various isomers, fluxionality, multiple reactive sites, and electronic charge density rearrangement upon interaction.^33−36^

In this context, considering the promising applications of Pt subnanoclusters in heterogeneous catalysis and the functional advantages of graphene as a stabilizing substrate, our study aims to elucidate the mechanisms behind molecular adsorption of Pt nanoclusters in the gas-phase and supported on graphene, using Sabatier’s principle as a guiding reference.^37,38^ Specifically, this study employs DFT calculations with D3 corrections to elucidate the adsorption mechanisms of CO, NO, N_2_, and O_2_ on Pt subnanoclusters supported by graphene substrates compared to gas-phase nanoclusters. By systematically exploring these interactions, we seek to enhance our understanding of how graphene-supported Pt subnanoclusters can be optimized for catalytic applications, advancing theoretical knowledge and practical applications in nanocatalysis.

## 2 Methodology

### 2.1 Computational Details and Theoretical Approach

All spin-polarized calculations were conducted by using DFT,^39,40^ employing the Perdew–Burke–Ernzerhof (PBE) exchange-correlation functional based on a generalized gradient approximation.^41^ To enhance accuracy, we included van der Waals (vdW) D3 corrections,^42,43^ which is a cheap and efficient approach concerning the inclusion of the long-range weak interactions.^44^ The DFT-PBE + D3 framework has shown reliable results in studies involving molecules, nanoclusters, and graphene.^36,45−48^

The Kohn–Sham (KS) orbitals were expanded using the projector augmented wave (PAW) method,^49,50^ as implemented in the Vienna *Ab initio* Simulation Package (VASP).^51,52^ In this method, core electrons are described fully relativistically, while valence electrons are treated by using a scalar-relativistic approximation.^53,54^ Further details on projectors are available in Table S1 of the Supporting Information (SI).

The KS orbitals were expanded in plane waves with a cutoff energy of 450 eV, which is 12.5% higher than the VASP recommendation. Calculations for free atoms, molecules (mol), isolated clusters (clu), molecules adsorbed on clusters (mol/clu), graphene (Gr) flakes, and clusters supported on Gr flakes (Gr-clu^(flake)^) were performed using a cubic box of 20 Å, ensuring a minimum separation of approximately 12 Å to avoid interactions between the system and its periodic three-dimensional (3D) images. For these systems, only a single ***k***-point (Γ-point) was used for Brillouin Zone (BZ) integration since these systems do not exhibit state dispersion in any BZ direction. The literature supports these parameters for similar systems using the same calculation methodologies.^36^ For systems involving periodic graphene substrates, including Gr, Gr with adsorbed clusters (Gr–clu), and molecules adsorbed on nanoclusters supported on Gr (Gr-(mol/clu)), a ***k***-mesh of 4 × 4 × 1 was employed for BZ integration, with a minimum separation of 17 Å along the *z*-axis to ensure a minimum distance between its periodic images.

In all optimizations, a small Gaussian smearing parameter of 1 meV was applied to prevent fractional occupation of electronic states. Equilibrium configurations were obtained based on a convergence criterion for Hellmann–Feynman forces of 0.010 eV/Å, representing structural relaxation and the system’s minimum energy state. The convergence criterion for electronic self-consistency was set to 1.0 × 10^–6^ eV. Vibrational frequencies (ν) were determined by calculating the dynamic (Hessian) matrix elements based on finite difference methods implemented in VASP. This involved two atomic displacements, with each atom moved in each direction by 0.01 Å. Further details on input parameters are available in the SI.

### 2.2 Atomic Configurations

**Figure 1:**
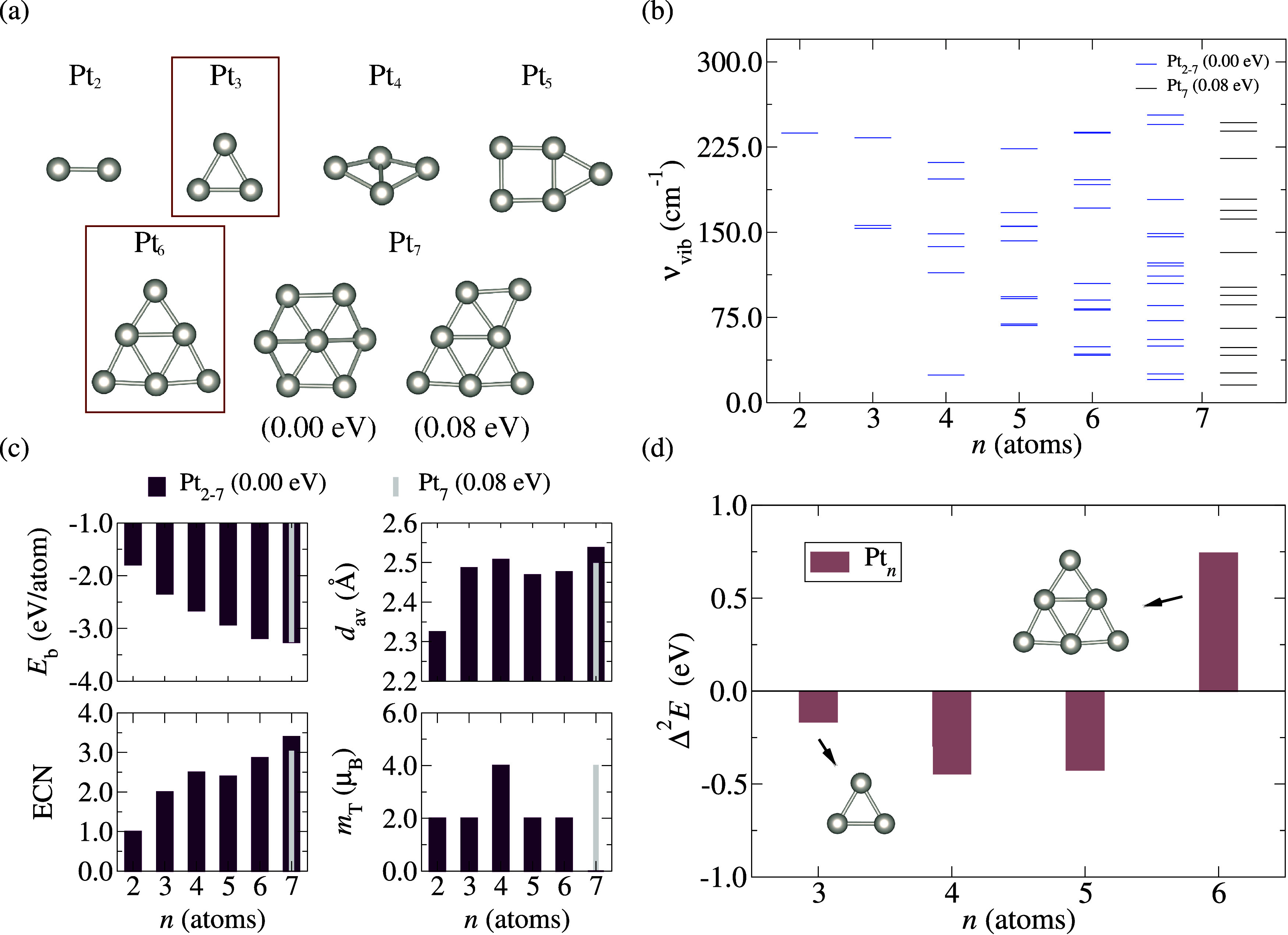
(a) The lowest energy structures of Pt_*n*_ (where *n* = 2–7 atoms), with emphasis on the most stable cluster sizes (*n* = 3 and 6), highlighted by brown rectangles. Also depicted are the two main isomers for Pt_7_ and their respective total energies. (b) Vibrational frequencies of the lowest energy configurations for Pt_*n*_ subnanoclusters. (c) Physicochemical properties of the lowest energy configurations of Pt_*n*_ systems as a function of the number of atoms, *n*: binding energy per atom (*E*_b_), average bond length (*d*_av_), effective coordination number (ECN), and total magnetic moment (*m*_T_). (d) Stability function, Δ^2^*E*, as a function of *n* for the lowest-energy Pt_*n*_ configurations, highlighting the two most stable sizes: Pt_3_ and Pt_6_.

To achieve the lowest energy configurations for Pt_*n*_ clusters (clu) (*n* = 2–7 atoms), as illustrated in Figure 1(a), we considered a diverse set of configurations, encompassing various geometries, including compact and open patterns, and one-, two-, and three-dimensional motifs. Different spin configurations were also explored, including ferromagnetic and antiferromagnetic options, following a strategy similar to our previous work.^55,56^

The lowest energy configurations of the Pt subnanoclusters were obtained from DFT-PBE + D3 optimizations without geometric constraints. Notably, Pt subnanoclusters generally favor planar configurations, and our results for *n* = 2–6 are consistent with the literature.^55,56^ However, for *n* = 7, the inclusion of vdW D3 corrections altered the most stable configuration, with our Pt_7_ configuration being 0.08 eV more stable than previously reported.^56^ The first high-energy isomers for Pt_7_ clusters had relative energy differences smaller than 11.5 meV per atom, which is less than *k*_B_*T* at room temperature, indicating the challenge of determining the putative global minimum configurations for these systems.

All the lowest energy configurations, including the higher Pt_7_ isomer, were confirmed as local minima through vibrational frequency analyses, exhibiting 3*N* – 6 (or 3*N* – 5 for linear geometries) only positive and real vibrational frequencies, as shown in Figure 1(b). The vibrational frequency eigenvalues and atomic coordinates for each structure are available in the SI. At the same time, the main physicochemical properties, e.g., binding energies per atom (*E*_b_), average bond length (*d*_av_), effective coordination number (ECN), and total magnetic moment (*m*_T_), of the lowest energy Pt_*n*_ nanoclusters as a function of *n* are presented in Figure 1(c).

For the subsequent molecular adsorption and support interaction studies, we selected the two most stable Pt subnanoclusters based on the stability function, Δ^2^*E*,^35,57^ defined as
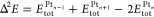
1where *E*_tot_^clu^ represents the total energy of the Pt subnanoclusters with *n* – 1, *n* + 1, and *n* atoms. Figure 1(d) illustrates the evolution of Δ^2^*E* for Pt_*n*_ subnanoclusters as a function of the number of Pt atoms. The most stable size was *n* = 6. However, considering possible entropic effects during nanocluster generation,^58^ which can produce many isomers and stability changes, we also considered the second most stable size, *n* = 3. Thus, we selected Pt_3_ and Pt_6_ subnanoclusters for further molecular adsorption and support interaction studies.

**Figure 2:**
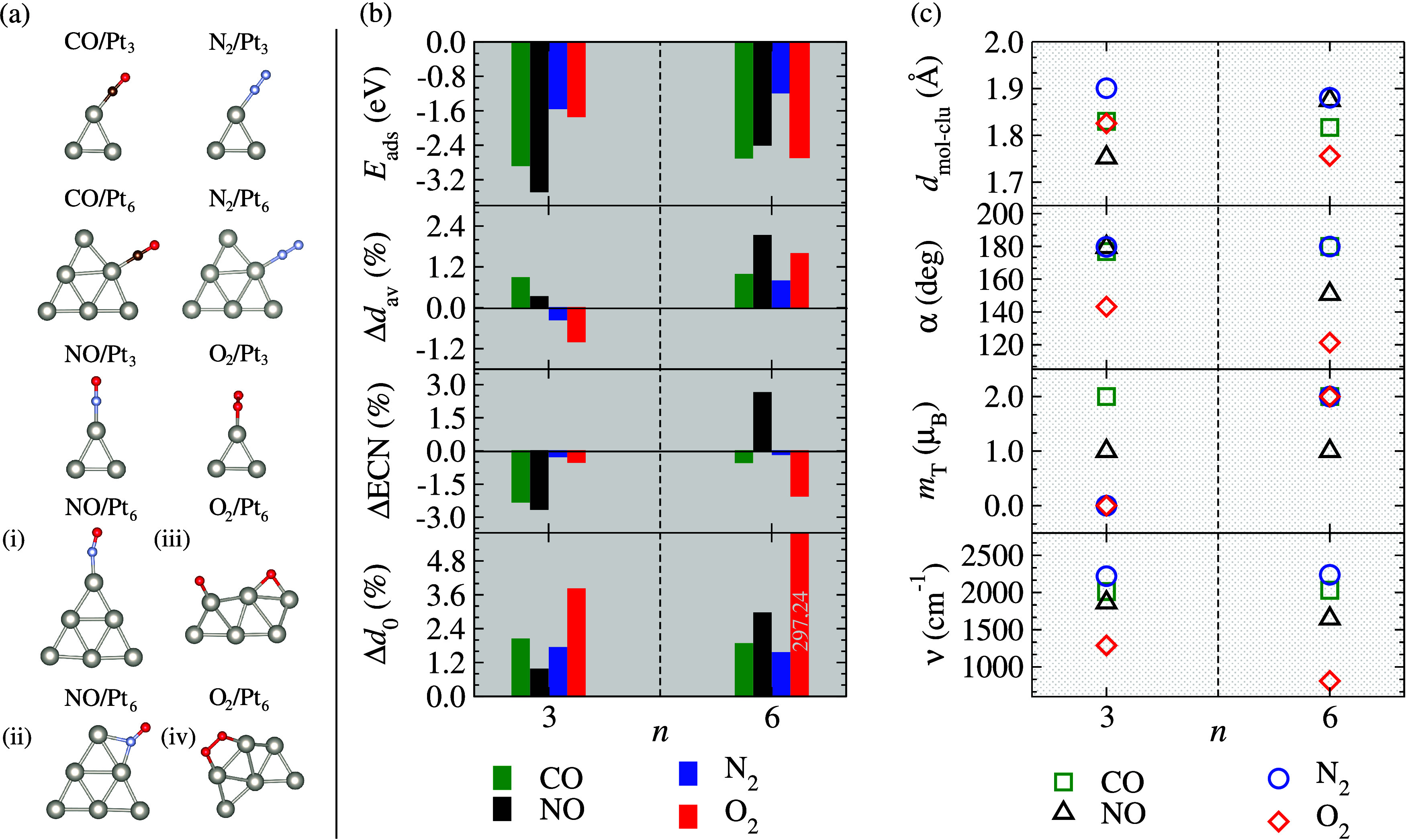
(a) The lowest energy adsorbed configurations for the CO/Pt_3,6_, N_2_/Pt_3,6_, NO/Pt_3,6_, and O_2_/Pt_3,6_ systems, including two additional structures for comparison. (b) Key adsorption properties of the lowest energy mol/clu systems: adsorption energy, *E*_ads_; relative deviation in the average bond length, Δ*d*_av_; relative deviation in the effective coordination number, ΔECN; and relative deviation in the equilibrium bond distance, Δ*d*_0_. (c) Additional properties include the minimum distance between the molecule and the cluster, *d*_mol-clu_; the molecular angle concerning the cluster, α; total magnetic moment, *m*_T_; and molecular vibrational frequencies ν.

For the molecular adsorption step, we considered four molecules (mol): CO, NO, N_2_, and O_2_, to be adsorbed on Pt_3_ and Pt_6_ subnanoclusters. We tested all nonequivalent adsorption positions, considering all possible onefold (top), 2-fold (bridge), and 3-fold (hollow) sites to identify the lowest energy adsorption sites. This procedure accounted for the different chemical environments in each nanocluster, from varying coordination numbers and molecular orientations. The lowest energy mol/clu configurations: CO/Pt_3,6_, NO/Pt_3,6_, N_2_/Pt_3,6_, and O_2_/Pt_3,6_, are shown in Figure 2(a), while the main properties of these adsorbed systems are depicted in Figure 2(b),(c).

For the Pt_3,6_ subnanoclusters supported on graphene (Gr), we determined the appropriate C–C bond distance and tested various *xy* dimensions of the Gr substrate supercells to ensure sufficient accommodation of the nanoclusters on the Gr surface, maintaining a minimum distance of 10 Å to avoid interaction with their periodic images. We selected (i) a nonperiodic Gr flake, i.e., a finite planar piece of graphene saturated by hydrogens at the edges, with dimensions of 10 × 12 Å^2^, and (ii) a periodic Gr sheet, represented by a supercell of 6 × 6 unit cells. A 20 Å vacuum region was included along the *z*-axis in both models to prevent interactions with periodic images.

To obtain the most stable atomic configurations for subnanoclusters supported on Gr (Gr–Pt_3,6_ and Gr–Pt_3,6_^flake^), we explored the analyte-substrate potential energy surface (PES), examining the molecular adsorption configurational space and identifying the most stable adsorbed configurations. We employed a strategy based on *Ab Initio* Molecular Dynamics (AIMD) simulations using the Born–Oppenheimer dynamics approach.^59^ The AIMD simulations started with atomic configurations having the Pt_3_ and Pt_6_ subnanoclusters in an arbitrary orientation relative to the substrate, followed by a simulated annealing (SA) process,^60^ from 300 to 0 K, using a Nosé–Hoover thermostat within an NVT ensemble,^61,62^ providing enough thermal energy for the nanoclusters to explore likely adsorption sites and then be frozen in the most stable adsorption sites. Our SA-AIMD simulations were run for 5 ps, with a time step of 1 fs, to generate the adsorbed snapshots (more details in SI, Figure S1). In addition to the final AIMD configuration, snapshots were collected throughout the simulated annealing process and subsequently structurally optimized through DFT-PBE + D3 calculations. Finally, we performed molecular adsorption on the resulting Gr–clu systems. We assessed all nonequivalent top, bridge, and hollow sites, allowing the molecules to interact with the nanoclusters within Gr–clu substrates.

### 2.3 Property Analyses

The analyses of structural properties were conducted for both supported and nonsupported systems using a combination of tools, namely the Visualization for Electronic and Structural Analyses (VESTA) software,^63^ and the concept of effective coordination.^64,65^ These tools enabled the determination of the main equilibrium bond lengths (*d*_0_), bond angles (α), average bond lengths (*d*_av_), minimum molecule-cluster distances (*d*_mol-clu_), effective coordination numbers (ECN), and relative deviations for individual and combined systems (Δ*d*_0_, Δ*d*_av_, and ΔECN). The relative deviations were considered to assess the structural distortions induced by molecular adsorption on nanoclusters, with *d*_av,ads_ and ECN_ads_ (molecules, *d*_0_) being obtained after removing the molecules (nanoclusters) from the analyses. This enabled the calculation of changes in *d*_av_, ECN, and *d*_0_ before adsorption, as well as in *d*_av,ads_, ECN_ads_, and *d*_0,ads_ after adsorption. The percentage difference between structural values before and after adsorption is given by
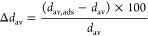
2
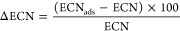
3and
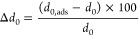
4

For energy analyses, we have used the relative total energy (Δ*E*_tot_^i^), defined as the difference between the total energy of an *i*-th configuration and the energy of the lowest-energy configuration, to classify the isomeric systems in order of stability. The binding energy per atom, *E*_b_, serves as an indicator of the stability of a molecule, nanocluster, or combined system, reflecting the energy gain experienced by each atom in a system composed of *n* atoms when they come together to form a cohesive structure. For a set of *n* atoms of a cluster, we have the expression: *E*_b_ = (*E*_tot_^clu^ – *nE*_tot_^Pt^) /*n*, where clu represents the selected Pt_*n*_ clusters, *E*_tot_^clu^ is the total energy of the cluster, and *E*_tot_^Pt^ is the total energy of a free Pt atom. For molecules, the binding energy per atom is given by *E*_b_^mol^ = (*E*_tot_^mol^ – *E*_tot_^X^ – *E*_tot_^Y^) /2, where mol = XY = N_2_, NO, CO, and O_2_, *E*_tot_^mol^ is the total energy of the molecule, and *E*_tot_^X^ or *E*_tot_^Y^ are the total energies of the isolated atomic species.

For adsorbed systems (nonsupported), both binding and adsorption energies were computed by considering the total energies of individual constituents and the interaction between molecules and nanoclusters.^36,47,66^ The binding energy per atom of the adsorbed system, *E*_b,ads_, is given by
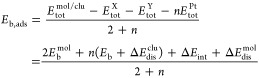
5while the adsorption energy, *E*_ads_, is defined as
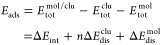
6where *E*_tot_^mol/clu^ represents the total energy of the mol/clu systems. Δ*E*_int_ is defined as the magnitude of the energetic contribution from the mol-clu interplay, without considering the energetic contribution from structural distortions. The equation gives this
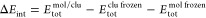
7Distortion energies, Δ*E*_dis_^clu^ (per atom) and Δ*E*_dis_^mol^ (per molecule), represent the energetic contribution from the relaxation energies upon adsorption. These are calculated as
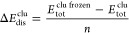
8and
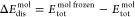
9where *E*_tot_^clu frozen^ and *E*_tot_^mol frozen^ represent the total energies of the frozen nanoclusters and molecules at their original positions within the mol/clu system whithout mol and clu parts, respectively. These values indicate the energy required to distort configurations from their initial to adsorbed stages.

For supported systems, Gr–Pt_*n*_ (*n* = 3 and 6), we calculated the adsorption energy per atom, *E*_ads_^Gr–Pt_*n*_^
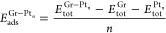
10and the binding energy per atom, *E*_b_^Gr–Pt_*n*_^:
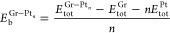
11where *E*_tot_^Gr–Pt_*n*_^ and *E*_tot_^Gr^ are the total energies for the Gr–Pt_*n*_ and Gr pristine systems, respectively. Finally, the adsorption energy per molecule for the mol on the Gr–clu systems, *E*_ads_^Gr-(mol/Pt)^, is given by
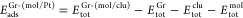
12where *E*_tot_^Gr-(mol/clu)^ represents the total energy of the molecules adsorbed on nanoclusters supported on Gr. Some extra details are provided in SI.

To deepen the understanding of the interaction energy’s physicochemical nature, we applied the Energy Decomposition Analysis (EDA) combined with the Natural Orbitals for Chemical Valence (NOCV), i.e., EDA-NOCV analysis,^67−74^ to investigate the mol···clu or mol···(Gr-clu) interactions. The computational details were based on DFT-PBE + D3, within the Zero-Order Regular Approximation,^75^ and an all-electron triple-ζ (TZ2P)^76^ basis set, as implemented in the Amsterdam Density Functional (ADF) software.^77−79^ From the EDA-NOCV analysis, the Δ*E*_int_ between the fragments was decomposed into physically significant terms
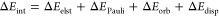
13where Δ*E*_elst_ represents the quasi-classical electrostatic interaction energy between the charge densities of the fragments, considering the frozen charge distribution; Δ*E*_Pauli_ denotes the Pauli exchange repulsion interaction between occupied orbitals of the fragments; Δ*E*_orb_ is the orbital interaction energy arising from the orbital mixing of the fragments, i.e., between occupied and unoccupied orbitals of interacting fragments (charge transfer) and within the same fragment (polarization); and Δ*E*_disp_ is the dispersion correction energy.

Finally, charge distribution and hybridization index analyses were conducted to elucidate the interaction mechanisms. The charge analysis was based on the Bader method.^80^ This method involves partitioning the atomic region into volumes known as Bader volumes, *V*_Bader_, based on charge density. Each volume *V*_Bader_ contains a unique maximum density and is separated by zero-flux surfaces *S*(**r**_s_),^81^ defined by the condition ∇*n*(**r**_s_)·S(**r**_s_) = 0, thereby delineating interatomic regions within each *V*_Bader_. The volume *V*_Bader_ = *V*_α,s_ around the atomic site α possesses an associated charge density, represented as
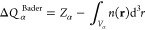
14where *Z*_α_ denotes the valence of the atomic site α. Several approaches can determine the volumes *V*_Bader_, with charges optimized through topologies such as Voronoi polyhedra.^82^

The hybridization index (hyb) was calculated qualitatively,^45,83^ to characterize the orbital contributions to the chemical bonding from the valence s, p, and d states. For example, the sd, sp, or pd hybridization can be accessed by the hybridization index,^84^ which is given for an *N*-atom system (cluster, molecule, graphene, or combined system) by
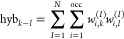
15where *k* and *l* are s, p, or d states with *k* ≠ *l*, *w*_*i*,*k*_^(*I*)^ (*w*_*i*,*l*_^(*I*)^) is the projection of the *i*-th KS orbital onto the *k* (*l*) spherical harmonic centered at atom *I.* hyb_*k*–*l*_ is composed of the nonzero contributions for the *k* and *l* local density of states for all atoms in the system. Thus, it is obtained by summing the product of the *k* and *l* contributions.

## 3 Results and Discussion

### 3.1 Gas-Phase Molecular Adsorption

#### 3.1.1 Nanoclusters and Molecules Properties

The lowest energy structures for Pt_*n*_, *n* = 2–7 atoms, shown in Figure 1(a), represent the most stable configurations resulting from a diversified configuration set search. The planar (two-dimensional) growth pattern for the most stable Pt nanoclusters, obtained from our scalar-relativistic DFT-PBE + D3 calculations, was confirmed as local minima through vibrational frequency analyses (Figure 1(b) and SI). The main physicochemical properties of the lowest energy configurations as a function of *n* are presented in Figure 1(c), specifically, binding energy per atom (*E*_b_), average bond length (*d*_av_), effective coordination number (ECN), and total magnetic moment (*m*_T_). As the number of atoms increases, there is an increase in the magnitude of *E*_b_, which tends toward the cohesive energy of bulk Pt (−5.84 eV),^85^ in agreement with previous work.^56^ We observed that *d*_av_ increases from *n* = 2 to 3, followed by small downward oscillations, maintaining similar *d*_av_ values between *n* = 3 and 7. This trend is directly related to structural changes imposed by different geometric configurations, showing deviation from a plateau corresponding to the planar pattern.

The ECN values increase almost linearly with the number of atoms, with a notable deviation for *n* = 5, which is associated with the presence of a square in the geometry as opposed to the basic triangular unit. The *m*_T_ values do not exhibit a well-defined pattern since the Pt electronic configuration (d^9^s^1^) is sensitive to minor structural changes, resulting in small orbital contributions. Consequently, the energy differences between ground- and metastable states for the *m*_T_ configurations are minimal, as observed for Pt_7_ isomers.^86^ Finally, Figure 1(d) shows the energetic selection among the lowest energy Pt_*n*_ subnanoclusters, where we selected Pt_6_ as the lowest energy size and Pt_3_ as the second most stable size for further study. These two subnanoclusters will be used for molecular adsorption and graphene-supported processes.

The main properties for N_2_, NO, CO, and O_2_ gas-phase molecules are shown in Table 1, which includes *E*_b_^mol^, *d*_0_, and ν. Our results exhibit good agreement with experimental and theoretical values from the literature,^36,87−92^ with the largest deviations from experimental results being 11.1% for *E*_b_^mol^, 1.8% for *d*_0_, and 4.2% for ν.^87−91^ The N_2_ and CO molecules exist in a singlet state with closed-shell electronic configurations, meaning all bonding orbitals are filled. Conversely, NO and O_2_ have open-shell configurations, where transferred electrons can occupy the half-filled antibonding orbitals. The NO molecule has an unpaired antibonding electron, while O_2_ exists in a triplet state with two unpaired electrons occupying two degenerate antibonding molecular orbitals.

**Table 1:** Main Molecular Properties: Binding Energies (*E*_b_^mol^), Equilibrium Bond Lengths (*d*_0_), and Vibrational Frequencies (ν) for N_2_, NO, CO, and O_2_ Gas-phase Molecules

mol	*E*_b_^mol^ (eV)	*d*_0_ (Å)	ν (cm^–1^)
N_2_	–5.20	1.11	2428
NO	–3.61	1.17	1922
CO	–5.75	1.14	2131
O_2_	–3.04	1.23	1570

#### 3.1.2 Molecular Adsorbed System Properties

We performed molecular adsorptions of N_2_, NO, CO, and O_2_ on the lowest energy subnanoclusters (Pt_3_ and Pt_6_), exploring all nonequivalent top, bridge, and hollow adsorption sites. Figure 2(a) shows the lowest energy configurations for these mol/clu systems, while Figure 2(b) presents their main adsorption properties: *E*_ads_, Δ*d*_av_, Δ*E*CN, and Δ*d*_0_. Figure 2(c) depicts *d*_mol-clu_, the molecule–subnanocluster angle (α), *m*_T_, and ν upon adsorption. In most cases, adsorption occurs predominantly at the onefold top site, except for NO/Pt_6_, where the bridge site–configuration (ii) in Figure 2(a) – exhibits slightly lower adsorption energy (0.01 eV) compared to the top site as depicted at configuration (i). For O_2_/Pt_6_, we observed complete dissociation of the O_2_ molecule and rearrangement within the nanocluster structure–configuration (iii). This phenomenon is directly related to electron transfer facilitated by the interaction between the d-orbitals of the Pt atoms and the antibonding π* orbitals of the O_2_ molecule (see Section 3.1.4). Additionally, configuration (iii) is 0.72 eV more stable than configuration (iv). Our analysis confirms that CO interacts via its C atom, NO via its N atom, while the O atom remains exposed to vacuum.

From Figure 2(b), a trend in *E*_ads_ shows stronger interactions for NO and CO molecules and weaker adsorption for N_2_ on both subnanoclusters. For O_2_, moderate adsorption occurs when only one O atom interacts via the top site, whereas molecular dissociation or dual O atom interaction intensifies adsorption (oxidation). Except for O_2_/Pt_6_ (molecular dissociation), |*E*_ads_| is higher for molecules adsorbed on the smaller Pt clusters (*n* = 3), reflecting their lower coordination and higher reactivity compared to *n* = 6 subnanoclusters, consistent with prior findings.^36^ Structural impacts are evident in deviations of *d*_av_ and ECN in nanoclusters, and *d*_0_ in molecules, upon adsorption. Δ*d*_av_ generally remains below 1.0%, except for NO/Pt_6_ and O_2_/Pt_6_, which show Δ*d*_av_ of 2.1 and 1.6%, respectively. Δ*E*CN values range between −2.6 and 2.6%. Relative deviations in *d*_0_ indicate expansions ranging from 1.0 to 2.9% for all adsorbed cases, with O_2_/Pt_3_ exhibiting a larger expansion (3.8%), and O_2_/Pt_6_ dissociating the molecule, causing a geometric transformation in the original Pt_6_ structure.

Figure 2(c) shows *d*_mol-clu_ values correlating with |*E*_ads_|, where larger *E*_ads_ magnitudes correspond to smaller distances between molecules and subnanoclusters. Molecule–nanocluster angles α approach 180° (176.9° ≤ α ≤ 179.9°), typical for linear top sites, except for NO/Pt_6_ and O_2_/Pt_3_ configurations showing tilted top-site orientations (151 and 143.2° for α, respectively), while O_2_/Pt_6_ does not measure α due to dissociation. Upon adsorption, the total magnetic moment is dominated by the nanocluster (*m*_T_ = 2.0 μ_B_) for closed-shell electronic molecules. For N_2_/Pt_3_, although the *m*_T_ = 2.0 μ_B_ configuration is slightly less stable than the *m*_T_ = 0 configuration, they are practically degenerate. For NO/clu systems, the magnetic moment is reduced to *m*_T_ = 1.0 μ_B_ due to the unpaired electron in NO. For O_2_/Pt_3_, the combination of the two unpaired electrons from O_2_ with the unpaired electrons from Pt_3_ results in a nullified total magnetic moment (*m*_T_ = 0). This explanation does not apply to O_2_/Pt_6_, where the dissociation of O_2_ alters the system’s magnetic properties. Finally, molecular vibrational frequencies align with gas-phase values, indicating expansions in equilibrium distances upon adsorption, reflective of weakened molecular bond strengths across all diatomic species adsorbed on subnanoclusters.

#### 3.1.3 Energetic Bias

**Figure 3:**
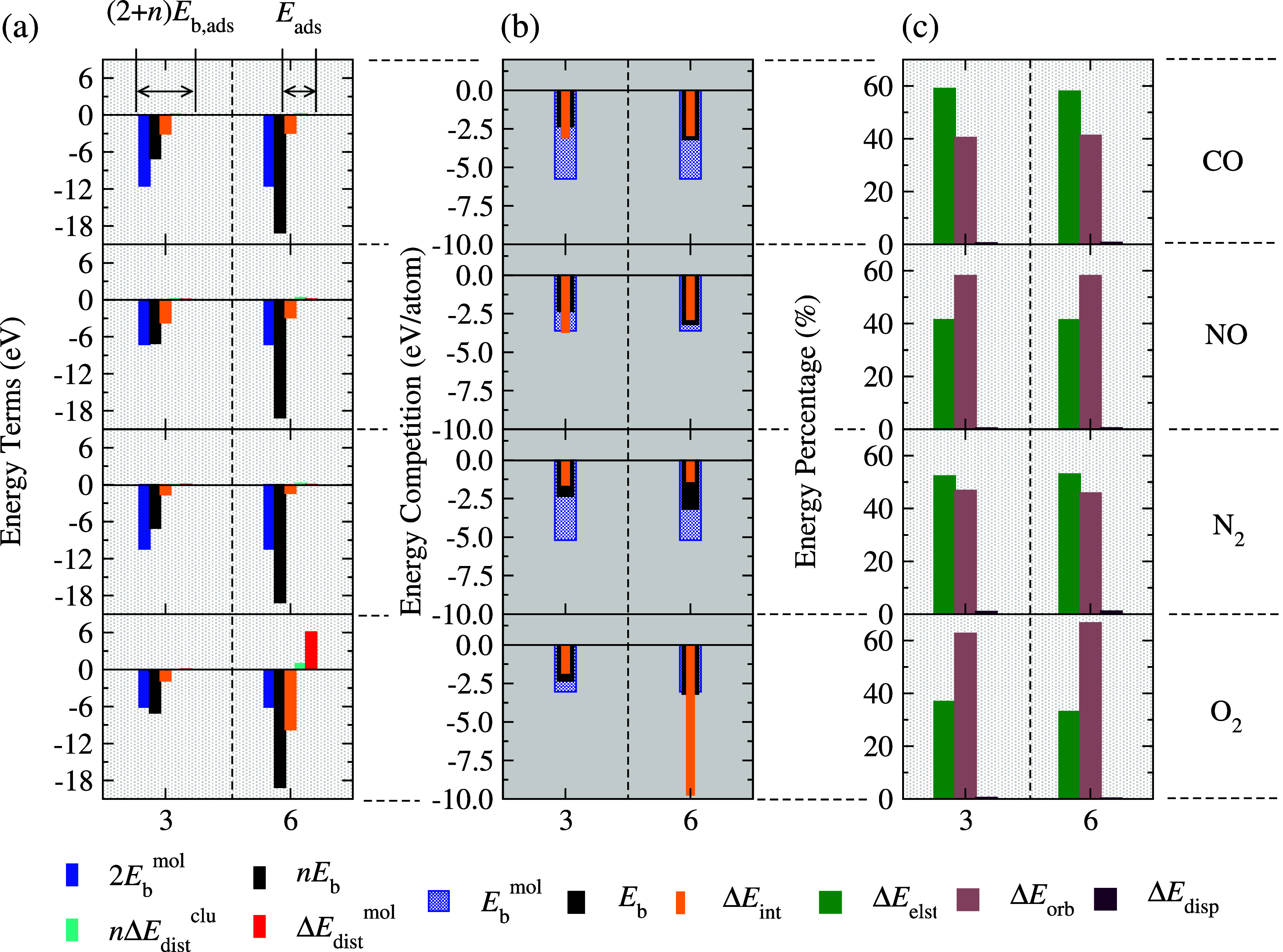
Energetic analysis of the lowest energy adsorbed systems: (a) energy components from (2 + *n*) *E*_b,ads_ and *E*_ads_, encompassing 2*E*_b_^mol^, *nE*_b_, Δ*E*_int_, *n*Δ*E*_dis_^clu^, and Δ*E*_dis_^mol^ terms; (b) energy competition among *E*_b_^mol^, *E*_b_, and Δ*E*_int_ (per atom); and (c) percentage contributions to Δ*E*_int_ from EDA, specifically Δ*E*_elst_, Δ*E*_orb_, and Δ*E*_disp_.

We conducted an energy analysis to elucidate the energetic trends of adsorbed systems in a gas-phase chemical environment for all lowest energy mol/clu systems, highlighting the clu–mol interaction strength. Figure 3 presents our findings: panel (a) decomposes the energetic terms contributing to the cohesion of complete adsorbed systems, emphasizing the significance of the interaction energy term (Δ*E*_int_). Panel (b) illustrates the competitive energy dynamics, showcasing our nanoclusters as promising candidates for these molecular interactions. Panel (c) provides an in-depth analysis of the interaction energies of the fragments (clu and mol), employing EDA to elucidate the dominant physical nature of these energetic terms. Further details on energy decomposition can be found in Table S2.

Examining the binding energy of mol/clu adsorbed systems according to eq 5, Figure 3(a) reveals that *E*_b,ads_ is primarily governed by the binding energies of the individual systems (clu and mol), with the subnanoclusters’ binding energy predominating for *n* = 6 systems, contrasting the less stable *n* = 3 nanocluster where the molecule’s binding energy becomes significant. The molecular adsorption process, characterized by *E*_ads_ (eq 6), highlights the crucial role of Δ*E*_int_, which is most pronounced for CO and NO, and weaker for N_2_. Nonadditive terms (*n*Δ*E*_dis_^clu^ and Δ*E*_dis_^mol^) show small energy discounts (0.02–0.36 eV) for CO-, NO-, and N_2_-adsorbed systems, reflecting minimal structural deformations upon adsorption. Notably, O_2_/Pt_6_ exhibits significant structural alterations and molecular dissociation due to strong clu–mol interactions, resulting in substantial nonadditive terms (0.97 and 6.08 eV, respectively).

According to Sabatier’s principle,^37,38^ an ideal catalyst exhibits moderate binding to adsorbates throughout the reaction intermediates (molecules) steps, striking a balance between weak nonbonding interactions and overly strong chemical bonds. A Δ*E*_int_ magnitude equal to or exceeding that of individual binding energies (*E*_b_ or *E*_b_^mol^) indicates either weak interaction (nonbonding) or excessively strong bonding, both detrimental to reaction rates.^47,48,93^ From Figure 3(b), we observe that CO/Pt_3_, NO/Pt_3_, and O_2_/Pt_6_ violate Sabatier’s principle, with Δ*E*_int_ exceeding one or both binding energies. Conversely, for other cases, |*E*_b_^mol^| > |*E*_b_|, and 0 < |Δ*E*_int_| < |*E*_b_|. Thus, Pt_3_ shows promise as a catalyst for N_2_ and O_2_ molecules, while Pt_6_ is favorable for N_2_, NO, and CO adsorptions. This criterion, however, does not consider kinetics or other experimental conditions.^94^

Further insights into Δ*E*_int_ from Figure 3(c) reveal that dispersive contributions are minimal (0.2–1.1%). The nature of mol–clu interactions is dominated by molecular electronic behavior, with N_2_···clu and CO···clu exhibiting electrostatic dominance and O_2_···clu and NO···clu showing orbital contributions. The contributions Δ*E*_elst_, Δ*E*_orb_, and Δ*E*_disp_ to Δ*E*_int_ have similar magnitudes across subnanocluster sizes: 52.7% Δ*E*_elst_, 46.3% Δ*E*_orb_, and 1.0% Δ*E*_disp_ for N_2_···clu; 58.5% Δ*E*_elst_, 40.9% Δ*E*_orb_, and 0.6% Δ*E*_disp_ for CO···clu; 34.9% Δ*E*_elst_, 64.7% Δ*E*_orb_, and 0.4% Δ*E*_disp_ for O_2_···clu; and 41.4% Δ*E*_elst_, 58.2% Δ*E*_orb_, and 0.4% Δ*E*_disp_ for NO···clu.

#### 3.1.4 Charge Analysis

**Figure 4:**
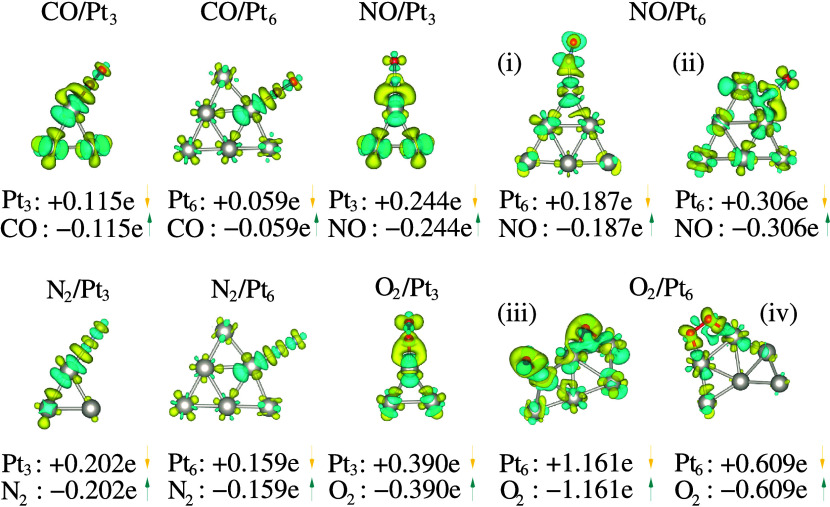
Bader charge analysis, Δ*Q*_Bader_, for the lowest energy CO/Pt_3,6_, N_2_/Pt_3,6_, NO/Pt_3,6_, and O_2_/Pt_3,6_ systems, illustrating charge losses (yellow) and gains (cyan) per atom. Effective charge transfers between clusters and molecules are also depicted, expressed in units of *e*. Isosurfaces are uniformly set to 0.006 for all cases.

The charge distribution analysis for the mol–clu systems is depicted in Figure 4. It reveals a net charge flux from subnanoclusters to molecules, resulting in anionic molecules (in cyan) and cationic nanoclusters (in yellow). This indicates a tendency for substrates to lose electron density upon interaction, while molecular species tend to gain electron density. This trend aligns with the Pauling electronegativity values: 2.28 for Pt, 2.55 for C, 3.04 for N, and 3.44 for O. The magnitude of electron density flux correlates with the electronegativity difference between Pt adsorption sites and the adsorption atoms (C, N, and O).

Across all systems, we observe moderate charge exchanges, except in cases with pronounced molecular interactions, such as O_2_/Pt_3_ (O_2_/Pt_6_), where a quasi-oxidation (oxidation) process occurs with significant net charge transfer from the Pt subnanoclusters to the O_2_ molecule. Notably, Figure 4 highlights specific cases, such as the increased charge flux for NO/Pt_6_ when changing the adsorption site from top (configuration (i)) to bridge (configuration (ii)), and the decreased charge flux for O_2_/Pt_6_ when O_2_ dissociates (configuration (iii)) compared to adsorption on double top sites (configuration (iv)), which introduces an additional channel for charge transfer. In the dissociation case, there is a substantial charge transfer from the nanocluster to the O_2_ molecule, amounting to 1.161*e*, where the excess charge populates the π* antibonding orbitals, leading to a decrease in the bond order between the oxygen atoms and weakening of the O–O bond.

The trend in charge density flow closely correlates with the equilibrium distance between the molecule and the nanocluster, as well as with the hybridization of the molecule’s s and p orbitals with the subnanocluster’s d orbitals (see hybridization indices in SI Table S5 for spin-polarized adsorption configurations of the most stable systems postadsorption). For instance, in mol/Pt_3_ systems, a longer *d*_clu–mol_ generally correlates with higher sp hybridization indices, indicating a less pronounced overlap between the molecule’s s and p orbitals and the nanocluster’s d orbitals. This reduced overlap results in lower charge transfer rates due to the weaker interaction. Conversely, systems with higher sd hybridization indices, which typically occur at shorter *d*_clu–mol_, demonstrate stronger interactions between the molecule’s s orbitals and the nanocluster’s d orbitals, leading to increased electron density transfer. In N_2_-based systems, the longer *d*_clu–mol_ leads to increased sp hybridization, as the greater distance reduces the overlap with the nanocluster states, thereby minimizing the overall hybridization with the cluster. This results in a lower charge transfer rate. On the other hand, NO-based systems exhibit shorter *d*_clu–mol_ values, which result in higher sd hybridization indices due to the strong interaction between the unpaired electron in the NO molecule’s π* orbital and the nanocluster’s d orbitals. This strong interaction is further enhanced by the partial radical character of the NO molecule, leading to more substantial charge transfer. CO and O_2_ systems exhibit intermediate *d*_clu–mol_ values, resulting in a balance between sp and sd hybridization indices, and displaying intermediate levels of hybridization and charge transfer. Thus, oxidative processes are influenced by significant electronegativity differences between interacting species and the hybridization of molecules’ s and p orbitals with nanoclusters’ d orbitals.^95^

In mol/Pt_6_ systems, the charge density flow trend correlates with the hybridization of molecules’ s orbitals with subnanoclusters’ d orbitals. Higher sd hybridization indices correspond to lower rates of electron density transfer, whereas lower sd hybridization indices correspond to higher charge transfer rates. This trend is directly linked to the equilibrium distance between the molecule and the nanocluster: shorter *d*_clu–mol_ values correspond to lower sp hybridization indices (e.g., O_2_/Pt_6_ system), while longer equilibrium distances correspond to higher hybridization indices between molecules’ s and p orbitals (e.g., N_2_/Pt_6_).

### 3.2 Supported Molecular Adsorption

#### 3.2.1 Supported Pt Nanoclusters

**Figure 5:**
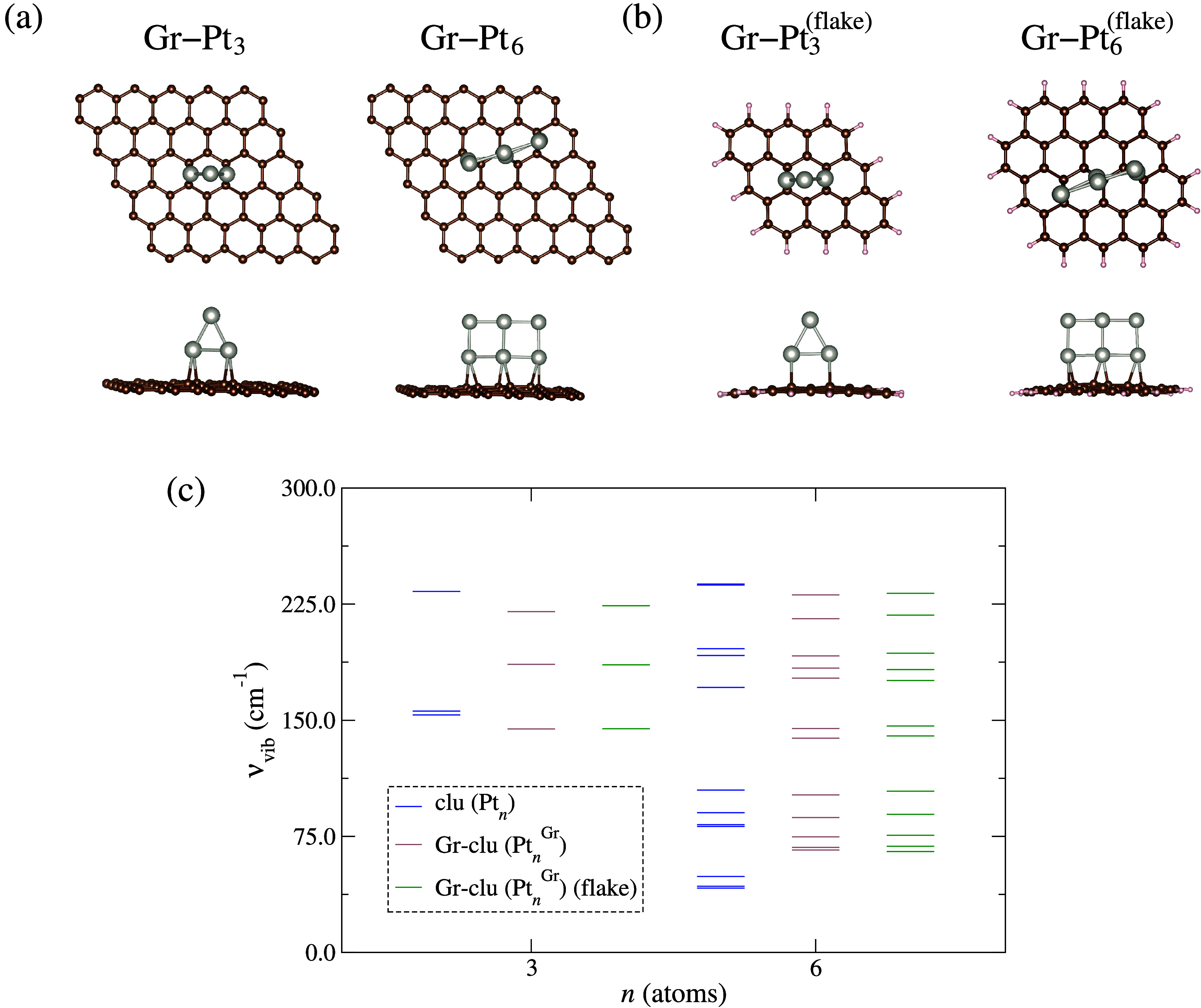
Lowest energy configurations of Gr–clu obtained from AIMD simulations after DFT-PBE + D3 optimization: (a) for Gr–Pt_3,6_ and (b) Gr–Pt_3,6_^flake^. (c) Vibrational frequencies (ν_vib_) for the Pt_3_ and Pt_6_ subnanoclusters in the gas-phase (blue), supported on periodic Gr (brown), and on nonperiodic (flake) Gr (green).

Following our previous study of clu–mol interactions in gas-phase nanoclusters, we investigate these interactions when the subnanoclusters are supported on Gr substrates. To ensure comprehensive analysis, we explore two models of Gr substrates: periodic and nonperiodic (flake). In Figure 5(a),(b), we present the lowest energy configurations of Gr–Pt_3,6_ and Gr–Pt_3,6_^flake^ systems, respectively. Panel (c) compares the vibrational frequencies (ν_vib_) of Pt clusters in gas-phase and supported on both Gr substrates.

The most stable Gr–clu configurations were identified from snapshots of the PES obtained via AIMD simulations, followed by structural optimization using the DFT-PBE + D3 protocol (described in the methods section). The most stable configurations of Pt_3_ and Pt_6_ deposited on the Gr substrate–panel (a) – are consistent with those on Gr flakes–panel (b) – and align with findings in the literature.^96,97^ Specifically, Pt subnanoclusters interact perpendicularly with Gr, with each nanocluster base atom bonding to bridge sites on the Gr nanosheets.

To assess the stability of these Gr-clu systems, we analyzed their vibrational spectra and compared them to the respective gas-phase subnanoclusters, as depicted in Figure 5(c). For the gas-phase subnanoclusters, all 3*N* – 6 vibrational frequencies are real and positive, confirming that these structures are at local minima on the potential energy surface. When these nanoclusters are adsorbed on both periodic and nonperiodic Gr substrates, the vibrational spectra exhibit reduced spread, indicating their interaction with the substrate. This interaction constrains translational and rotational motions, effectively converting these into additional vibrational modes. Consequently, the adsorbed nanoclusters can theoretically exhibit up to 3*N* vibrational frequencies. However, for comparative purposes, we focused on the 3*N* – 6 vibrational modes, which are also real and positive, to maintain consistency with the gas-phase analysis. The reduced vibrational spread in the supported systems reflects the binding to the substrate, leading to decreased reactivity and enhanced stabilization of the Pt subnanoclusters on Gr substrates.

To deepen our understanding, we examined key properties of these Gr–clu systems, summarized in Table 2. Comparison between Pt subnanoclusters supported on periodic and nonperiodic Gr substrates reveals similar behavior in energetic (*E*_ads_^Gr–Pt_*n*_^ and *E*_b_^Gr–Pt_*n*_^), structural (*d*_av_^Gr–Pt_*n*_^ and ECN^Gr–Pt_*n*_^), and magnetic properties (*m*_T_^Gr–Pt_*n*_^).

**Table 2:** Main Gr–clu Properties: Binding Energies (*E*_b_^Gr–Pt_*n*_^), Adsorption Energies (*E*_ads_^Gr–Pt_*n*_^), Average Bond Lengths for Nanoclusters (*d*_av_^Gr–Pt_*n*_^), Effective Coordination Number for Subnanoclusters (ECN^Gr–Pt^_^*n*^_), and Total Magnetic Moment (*m*_T_^Gr–Pt_*n*_^)

	Gr–Pt_3_	Gr–Pt_6_	Gr–Pt_3_^flake^	Gr–Pt_6_^flake^
*E*_ads_^Gr–Pt_*n*_^ (eV)	–0.681	–0.399	–0.631	–0.396
*E*_b_^Gr–Pt_*n*_^ (eV)	–3.026	–3.584	–2.976	–3.581
*d*_av_^Gr–Pt_*n*_^ (Å)	2.482	2.481	2.485	2.480
ECN^Gr–Pt_*n*_^	1.994	2.333	1.993	2.333
*m*_T_^Gr–Pt_*n*_^ (μ_B_)	0.000	2.000	0.000	2.000

Nanoclusters experience slightly stronger stabilization when supported on periodic substrates, reflected in higher adsorption energy magnitudes. Notably, for the more reactive Pt_3_ cluster, we observed more significant differences in adsorption behavior depending on the substrate. As expected, Pt_3_ subnanoclusters, being smaller and less stable in the gas-phase, exhibit greater interaction energies (*E*_ads_^Gr–Pt_*n*_^) as compared to Pt_6_ systems. Specifically, *E*_b_^Gr–Pt_*n*_^ shows a larger increase for Pt_3_ subnanoclusters on periodic and nonperiodic Gr substrates (29.1 and 27.0%, respectively) when compared to gas-phase systems. For Pt_6_ subnanoclusters, this increase is 12.6 and 12.5%, respectively.

Structurally, nanoclusters exhibit similar characteristics on both support models, with Pt_3_ showing slight contractions (−0.185 and −0.064%) and Pt_6_ showing expansions (0.157 and 0.149%) in average bond lengths (*d*_av_^Gr–Pt_*n*_^) for periodic and nonperiodic substrates, respectively, relative to the gas-phase systems. Regarding the ECN^Gr–Pt_*n*_^, both Pt_3_ and Pt_6_ subnanoclusters show decreases, with Pt_3_ exhibiting a smaller decrease (−0.3%) and Pt_6_ a larger one (−18.5%) for both substrates compared to gas-phase. In terms of *m*_T_^Gr–Pt_*n*_^, Pt_3_ subnanoclusters reduce their magnetic moment from 2 to 0 μ_B_ on both supports, while Pt_6_ systems maintain an unchanged magnetic moment.

Notably, Pt_6_ subnanoclusters undergo a motif change upon adsorption on Gr substrates, transitioning from a basic triangular-unit structure in gas-phase to a double square structure, resulting in an atypical expansion of average bond lengths due to increased Gr–Pt_6_ interaction energy. Conversely, the interaction between Pt_3_ and the Gr substrate, while less pronounced, does not alter the structural motif but completely suppresses the total magnetic moment after adsorption.

These observations highlight two critical points: first, less stable or metastable nanoclusters in the gas phase can significantly increase stability when interacting with substrates such as Gr. Second, the equivalence in using periodic and nonperiodic Gr substrates for Pt subnanoclusters supports our findings, demonstrating consistent trends in their interaction behavior. Henceforth, we present results for molecular adsorption on supported nanoclusters considering periodic Gr substrates. Results for molecules adsorbed on subnanoclusters supported on Gr flakes are presented in Figure S2, with corresponding comparative properties in Table S4 of the SI.

#### 3.2.2 Molecular Adsorbed Supported-System Properties

**Figure 6:**
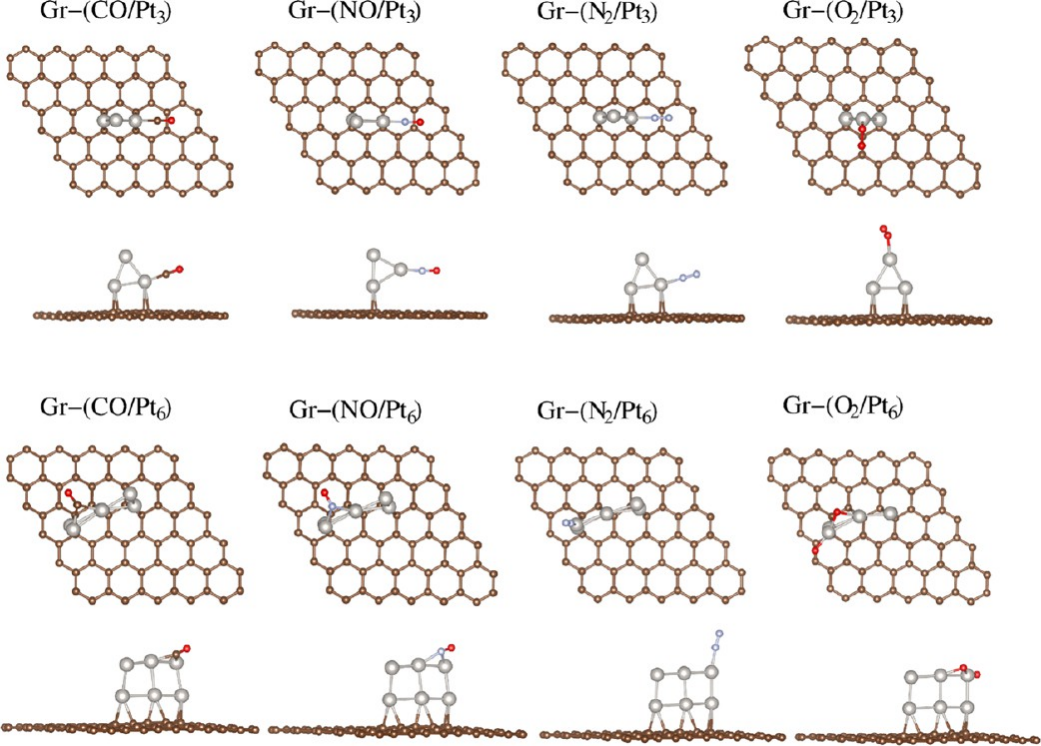
Lowest energy adsorbed configurations of the Gr–CO/Pt_3,6_, Gr–NO/Pt_3,6_, Gr–N_2_/Pt_3,6_, and Gr–O_2_/Pt_3,6_ systems, featuring top and side views for each configuration.

**Figure 7:**
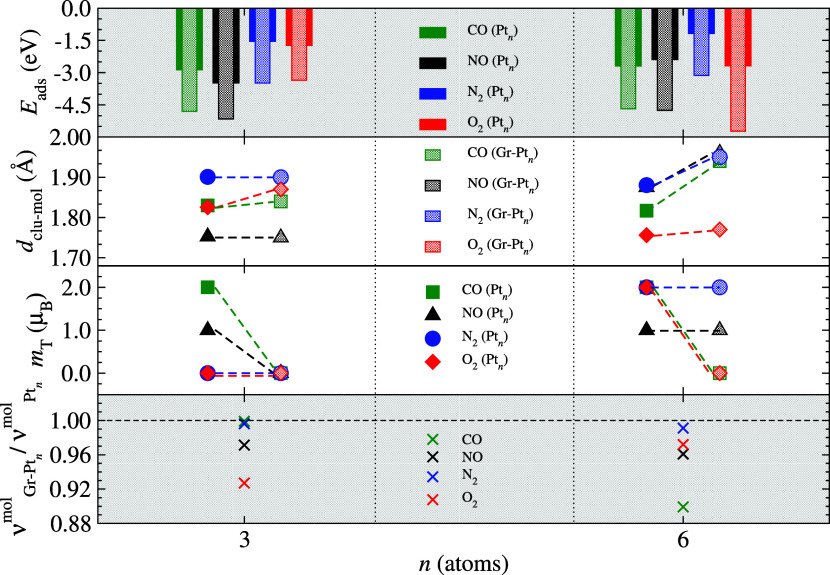
Main adsorption properties of the lowest energy mol/clu (symbols based on solid colors) and Gr-(mol/clu) (symbols based on transparent colors) systems: adsorption energy, *E*_ads_, distance between the molecule and the cluster, *d*_mol-clu_, total magnetic moment, *m*_T_, and vibrational frequencies, ν_vib_ (ratios of molecular modes after adsorption for the two substrates). Solid colors represent values in the gas-phase (mol/clu), while hatched patterns represent values obtained for supported systems, Gr-(mol/clu).

The adsorptions of N_2_, NO, CO, and O_2_ were performed on the lowest energy Gr–Pt_3_ and Gr–Pt_6_ systems, testing all nonequivalent top, bridge, and hollow adsorption sites. The lowest energy adsorbed configurations for the Gr-(mol/clu) systems are shown in Figure 6. The main adsorption properties for these configurations are depicted in Figure 7, including *E*_ads_, *d*_mol-clu_, *m*_T_, and ν_vib_ (molecular modes ratio after adsorption). Predominantly, adsorption occurs at the onefold top site for all cases, except for (i) Gr-(CO/Pt_6_) and Gr-(NO/Pt_6_) where adsorption occurs at the 2-fold bridge site, and (ii) Gr-(O_2_/Pt_6_), where complete dissociation of the O_2_ molecule is observed.

Similar to nonsupported systems, in the CO- and NO-based systems, molecular interaction with the nanoclusters occurs through the C and N atoms, respectively, with the O atom remaining exposed to vacuum. The N_2_-based system exhibits the least interaction, while the O_2_-based system may display oxidation characteristics, particularly in the Gr-(O_2_/Pt_6_) configuration. Thus, in Gr-(mol/Pt_3_) systems, the molecules interact with the substrates, forming adsorption angles (the angle between molecule atoms and Pt adsorption site) close to 180° (ranging from 176.6 to 179.4°), except for the Gr-(O_2_/Pt_3_) system, where the molecule is adsorbed at an angle of 129.1°. In Gr-(mol/Pt_6_) systems, the bridged adsorptions of CO and NO form angles of 139.8 and 130.3°, respectively, while the adsorption of N_2_ forms an angle of 173.3°. For Gr-(O_2_/Pt_6_), the angle is not measured, as the molecule is dissociated.

From Figure 7, we observe that for both subnanocluster sizes, the supported systems exhibit higher *E*_ads_ magnitudes compared to their gas-phase counterparts. This indicates a promising synergistic effect of nanoclusters deposited on graphene supports, where the intensified molecular interaction in the Gr–clu systems is supported by the increased stabilization of the subnanoclusters. Between the two sizes of nanoclusters, the *E*_ads_ magnitude of Pt_6_-based systems is slightly smaller than that of Pt_3_-based systems, a result stemming from the greater reactivity of smaller subnanoclusters. For systems with adsorbed O_2_, we observed a reduction in interaction with Gr–Pt_3_, in contrast to the significant intensification observed with Gr–Pt_6_, which assumes the role of the system with the greatest adsorption energy magnitude, even leading to the molecular dissociation of O_2_. CO and NO remain cases of intermediate adsorption, suitable for application in Gr–clu systems.

Regarding *d*_mol-clu_ values, we observed either maintenance or increased distances between the molecule and nanoclusters when transitioning from gas-phase to supported systems. Increases in *d*_mol-clu_ are directly linked to the intensified Gr–clu interaction. For *m*_T_, there is complete suppression of values for all Gr-(mol/Pt_3_) cases, provided that dangling bonds are satisfied. In contrast, for Gr-(mol/Pt_6_) systems, this suppression occurs for CO- and O_2_-based systems due to a bridge bond and molecular dissociation, respectively, while for NO and N_2_, the *m*_T_ behavior observed in the gas-phase is maintained. Finally, regarding molecular vibrational frequency, the ratio ν_Gr–Pt_*n*__^mol^/ν_Pt_*n*__^mol^ generally shows a reduction in values for molecules adsorbed in supported systems, corroborating the more intense molecular adsorption in these supported systems.

#### 3.2.3 Interaction Energy Analysis

To deepen our understanding of the molecular interaction on supported subnanoclusters, specifically the lowest energy Gr-(mol/Pt_*n*_) systems, we conducted two levels of energy decomposition analysis considering the interaction energy. The first level considers pairwise interactions, decomposing the Gr-(mol/Pt_*n*_) interaction energy as follows
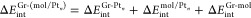
16where Δ*E*_int_^Gr–Pt_*n*_^, Δ*E*_int_^mol/*Pt*_*n*_^, and Δ*E*_int_^Gr–mol^ represent the interaction energies between the Gr substrate and Pt_*n*_ subnanoclusters, the molecules and Pt_*n*_ subnanoclusters, and the Gr substrate and molecules, respectively. The term Δ*E*_int_^Gr–mol^ is expected to provide a minor contribution, considering the possible interactions and distortions caused by the presence of the molecule on the Gr surface. Thus, by neglecting the Gr–mol interaction term, we can qualitatively evaluate and quantitatively estimate the term with the greatest contribution to the Gr-(mol/clu) interaction.

**Figure 8:**
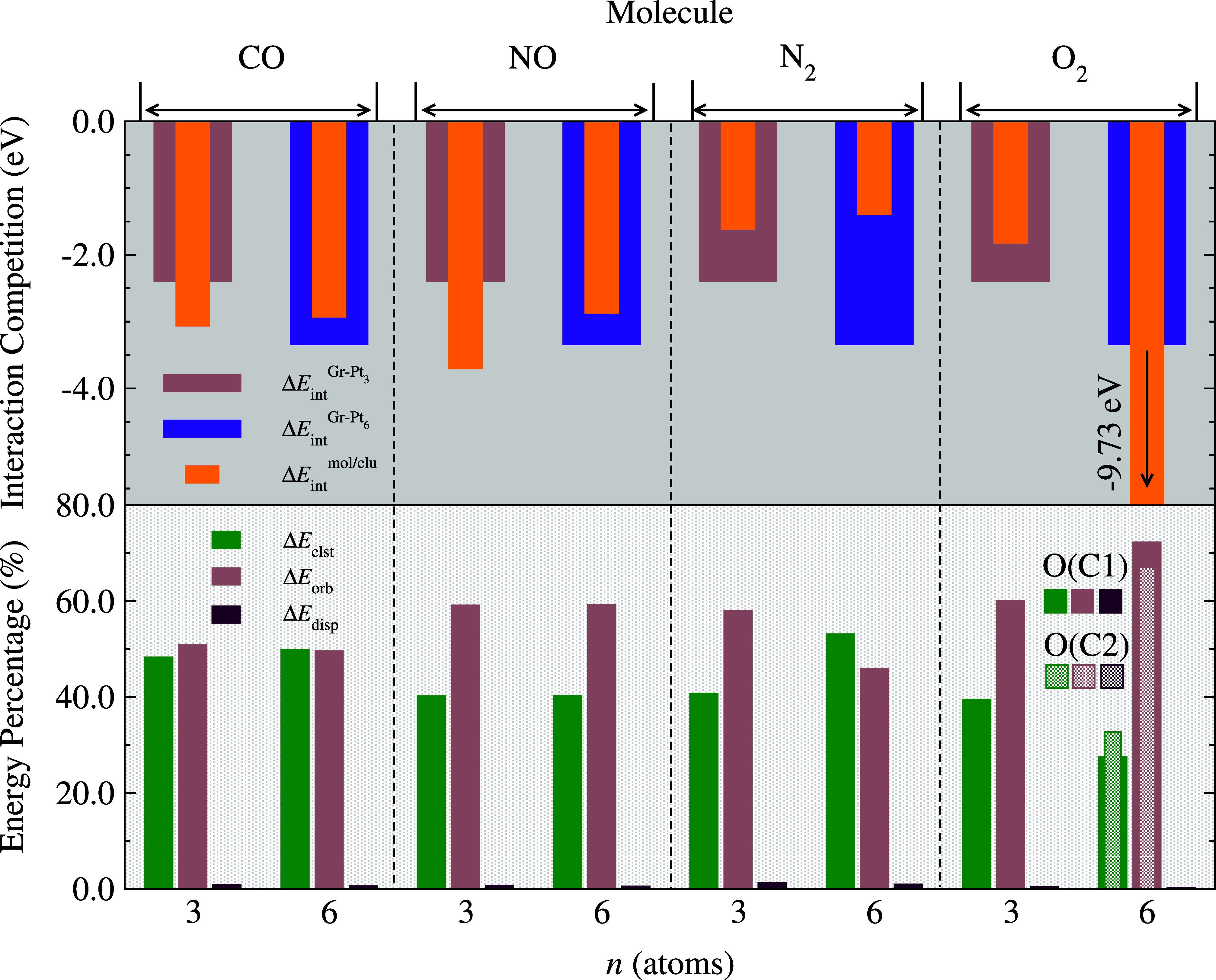
Energetic analysis of the lowest energy adsorbed Gr-systems: at the top, the competition in interaction energies for Gr-(mol/Pt_3_) in brown, Gr-(mol/Pt_6_) in magenta, and mol–clu in orange. At the bottom, the percentage contributions to Δ*E*_int_ from EDA: Δ*E*_elst_ (green), Δ*E*_orb_ (brown), and Δ*E*_disp_ (purple). For Gr-(O_2_/Pt_6_), interaction components are shown considering two different interaction sites after molecular dissociation, indicated by O(C1) and O(C2).

In Figure 8, top panel, we show the interaction strength for Gr–clu and mol/clu (clu = Pt_3,6_) terms within the supported chemical environments (see also Table S3). Regardless of the adsorbed molecular type, we observed similar Δ*E*_int_^Gr–Pt_*n*_^ values for all Gr–Pt_3_ cases, averaging around −2.39 eV, and for all Gr–Pt_6_ cases, averaging around −3.34 eV. The energetic differentiation for each molecular adsorption case is primarily due to the Δ*E*_int_^mol/*Pt*_*n*_^ term, which varies between −3.70 and −1.61 eV for Pt_3_-based systems, and between −9.73 and −1.39 eV for Pt_6_-based systems. As discussed previously, we have |Δ*E*_int_^Gr–Pt_6_^ | > |Δ*E*_int_^Gr–Pt_3_^|, corroborated by the structural transformation of the original Pt_6_ motif.

For the Δ*E*_int_^mol/*Pt*_*n*_^ term concerning Gr-(mol/Pt_3_) systems, we observe that the interactions involving CO and NO molecules with Pt_3_ are predominant over the Gr–clu interaction, categorizing these systems within the chemisorption context, with potential undocking of the mol/clu system. Conversely, the N_2_ and O_2_ molecules showed |Δ*E*_int_^mol/Pt_3_^| < |Δ*E*_int_^Gr–Pt_3_^|, ensuring the maintenance/recyclability of the catalyst (Gr–clu), while maintaining molecular adsorption at appreciable values for the chemical reaction. On the other hand, in the Gr-(mol/Pt_6_) case, we observe a different scenario where |Δ*E*_int_^mol/Pt_6_^| < |Δ*E*_int_^Gr–Pt_6_^| for mol = CO, NO, and N_2_, with N_2_ showing the weakest mol–clu interaction. This indicates Pt_6_’s potential as a good catalytic system for molecular interaction, where the Gr–clu interaction dominates clu–mol, ensuring the integrity of the catalytic system during molecular interaction. The only exception is for Gr-(O_2_/Pt_6_), where the interaction between O_2_ and Pt_6_ is significantly stronger, leading to molecular dissociation and potential oxidation, akin to catalytic poisoning.

Consistent with previous results for gas-phase cases, we confirm that the supported Pt_3_-based system is promising as a potential catalyst for N_2_ and O_2_ molecules, while the supported Pt_6_-based system is suitable for N_2_, NO, and CO adsorptions. To further understand Δ*E*_int_, Figure 8, bottom panel, presents an EDA-NOCV analysis at the interaction energy of the fragments, namely: (Gr-clu) and mol, aiming to identify the dominant physical nature of the energetic terms for molecular interaction relative to supported subnanoclusters. Similar to gas-phase cases, the Δ*E*_disp_ term has a very small percentage contribution. The molecular interaction with the Gr–clu substrate is dominated by the orbital term, indicating a predominance of covalent interaction for all systems, except Gr-(CO/Pt_6_) and Gr-(N_2_/Pt_6_), where the interaction nature is still dominated by the molecular electronic behavior, governed by the electrostatic term due to the molecular closed-shell configurations. Therefore, the Gr support plays a crucial role in stabilizing the nanoclusters and enhancing the covalent character of the mol–(Gr-clu) interaction.

We verify Δ*E*_elst_, Δ*E*_orb_, and Δ*E*_disp_ contributions with average values of 40.1% for Δ*E*_elst_, 59.1% for Δ*E*_orb_, and 0.8% for Δ*E*_disp_ for Gr-(N_2_/Pt_3_), Gr-(NO/Pt_6_), and Gr-(O_2_/Pt_6_). For Gr-(CO/Pt_3_), the contributions are 48.3% for Δ*E*_elst_, 50.8% for Δ*E*_orb_, and 0.9% for Δ*E*_disp_. For Gr-(CO/Pt_6_), the electrostatic and orbital contributions are nearly equal: 49.8% for Δ*E*_elst_, 49.6% for Δ*E*_orb_, and 0.6% for Δ*E*_disp_. For Gr-(N_2_/Pt_6_) and Gr-(NO/Pt_6_), the values are 53.1 and 40.2% for Δ*E*_elst_, 45.9 and 59.2% for Δ*E*_orb_, and 1.0 and 0.6% for Δ*E*_disp_, respectively. Finally, for Gr-(O_2_/Pt_6_), where molecular dissociation occurs, the values are shown separately for the interaction of the two O atoms (O(C1) and O(C2) cases), revealing average values of 30.1% for Δ*E*_elst_, 69.7% for Δ*E*_orb_, and 0.2% for Δ*E*_disp_, confirming the highest interaction values due to the covalent nature of O adsorption, typically resulting from the nanocluster oxidation process.

#### 3.2.4 Charge and Hybridization Analysis

**Figure 9:**
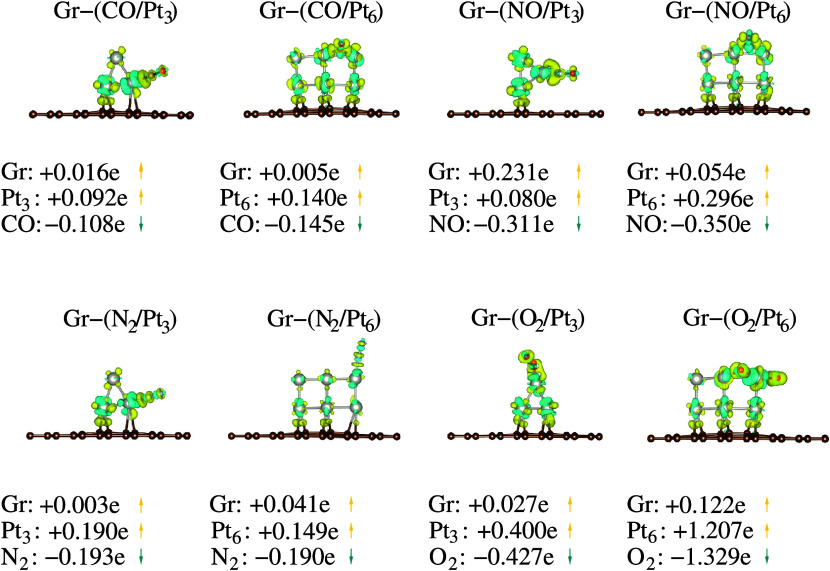
Bader charge analysis, Δ*Q*_Bader_, for the lowest energy Gr-(CO/Pt_3,6_), Gr-(NO/Pt_3,6_), Gr-(N_2_/Pt_3,6_), and Gr-(O_2_/Pt_3,6_) systems. Yellow indicates charge losses per atom, while cyan indicates gains per atom. Nominal values of effective charge transfers between clusters and molecules are also depicted in units of *e*. Isosurfaces for all cases are set to 0.009.

Additionally, we conducted a Bader charge analysis, as illustrated in Figure 9, to show the charge distribution upon adsorption for the Gr–(mol/clu) systems. As discussed earlier, we observed that the relationship between electronegativity and the tendency for electron density flow is also evident in the charge transfer between molecules and supported subnanoclusters. A final charge flux was observed from the Gr substrate and nanoclusters to the molecules, leading to anionic molecules and cationic Gr–clu substrates. The substrates lose electron density (indicated in yellow) upon interaction, while the molecular species gain electron density (indicated in cyan). For the most pronounced molecular interactions, the quasi-oxidation (Gr-(O_2_/Pt_3_)) and oxidation (Gr-(O_2_/Pt_6_)) cases showed a net charge transfer from the Gr substrates and Pt subnanoclusters to the O_2_ molecule.

Furthermore, the charge transfer rate does not show a direct dependency on the size of the Pt subnanoclusters. However, it is correlated to the hybridization of nanocluster orbitals with Gr sheets and molecular ones, which can imply different molecular adsorption behaviors. The hybridization indices (sd, sp, and pd) are presented in nominal values, considering the spin-polarized configurations of the lowest energy Gr-(mol/clu) systems in the SI (Table S6). Once again, we observe the relationship between the transferred charge densities and the pd hybridizations between the molecule and nanocluster orbitals, where higher (lower) hybridization indices indicate lower (higher) charge transfer rates from Gr–clu to the molecule. Overall, we note an increase in the charge transfer rates in Gr-supported systems compared to gas-phase adsorbed systems, except for the Gr-(CO/Pt_3_) and Gr-(N_2_/Pt_3_) systems, which showed reductions in transfer rates. The limiting case, characterized by the interaction of the O_2_ molecule with the Gr–Pt_6_ substrate, resulted in high charge transfer (oxidation) and consequently lower hybridization indices due to molecular dissociation.

For the Gr-(mol/Pt_3_) systems, we observe a correlation between the sd hybridization indices, charge density flow, and the *E*_ads_ values. Higher sd hybridization indices are associated with lower charge transfer rates and higher |*E*_ads_| values, while lower indices correspond to higher charge transfer rates and lower |*E*_ads_| values. For Gr-(mol/Pt_6_) systems, higher *sd* hybridization indices are associated with lower charge transfer rates and lower |*E*_ads_| values, while lower indices correspond to higher transfer rates and higher |*E*_ads_| values.

## 4 Discussion and Conclusions

This investigation provides a detailed analysis of the physicochemical properties and molecular adsorption mechanisms of CO, NO, N_2_, and O_2_ molecules on Pt_3_ and Pt_6_ nanoclusters using DFT-PBE + D3 calculations. We specifically focused on these clusters after identifying them as the most stable configurations in the gas phase (Pt_*n*_, where *n* = 2–7). Our study also explored the interactions of these nanoclusters with graphene substrates, in both periodic and nonperiodic forms, examining their structural, magnetic, and adsorption properties.

Following the identification of Pt_3_ and Pt_6_ as the most stable gas-phase nanoclusters, we transitioned to systems where these clusters were supported on graphene substrates. Our analysis, including Born–Oppenheimer AIMD simulations, provided insights into the stability and potential catalytic applications of these Gr-supported subnanoclusters. We observed that Pt_3_ exhibited higher reactivity, with larger binding energy magnitude and distinct electronic structures, while Pt_6_ demonstrated greater stability.

Our results confirmed that Pt subnanoclusters adhere to graphene substrates in stable configurations, with adsorption energies indicating stronger interactions and increased stabilization on periodic substrates compared to nonperiodic ones. Structural analyses revealed minor contractions in Pt_3_ and expansions in Pt_6_ upon adsorption, with a significant reduction in magnetic moments for Pt_3_ nanoclusters.

In the mol/clu systems, Pt_3_ exhibited higher adsorption energies and Bader charge transfer compared to Pt_6_, suggesting greater reactivity in the smaller cluster. After adsorption, vibrational frequencies generally decreased, indicating stability. Notably, O_2_ adsorption on Pt_6_ led to molecular dissociation, indicating chemisorption.

Our energy decomposition analysis highlighted the contributions of various interaction components between graphene substrates, Pt subnanoclusters, and adsorbed molecules, emphasizing the substrate’s role in modulating molecular interactions. The interaction energy emerged as a distinctive criterion to classify systems as ideal catalysts, in line with the Sabatier principle. Interaction energy values close to zero indicate minimal interaction, characterizing physisorption; while values larger than the binding energy magnitudes of constituent systems indicate powerful interaction, compromising desorption rates and catalyst reuse. Complementary, the hybridization indices between molecule and nanocluster orbitals are linked to charge density flow tendencies.

Based on our findings, we conclude that graphene-supported Pt_3_ is particularly effective for the adsorption of CO and NO, exhibiting higher binding energies and charge transfer, which suggests enhanced reactivity. On the other hand, Pt_6_ is more stable and better suited for O_2_ adsorption, where molecular dissociation is observed, indicating strong chemisorption. For N_2_ adsorption, while both clusters show weaker interactions compared to other molecules, Pt_3_ still demonstrates a slight edge in reactivity.

These findings provide important insights into molecular adsorption mechanisms on Pt subnanoclusters, which are critical for advancing theoretical and experimental studies in nanocatalysis. Since the interaction between CO, NO, N_2_, and O_2_ molecules and Pt_3_/Pt_6_ clusters on graphene substrates resulted in enhanced adsorption energies and stability compared to their gas-phase counterparts, highlighting the synergistic effect of the graphene substrate. Energy decomposition analysis emphasized the substrate’s significant role in modulating molecular interactions, leading to notable stabilization, particularly for NO and CO. Our findings underscore the importance of graphene as a substrate in enhancing the stability and catalytic properties of Pt subnanoclusters, with similar interaction trends observed for both periodic and nonperiodic graphene models.

Our study lays the groundwork for future research focused on optimizing the catalytic performance of graphene-supported Pt subnanoclusters, with further exploration of cluster sizes, shapes, compositions, and substrates promising deeper insights. An important future direction is to integrate our simulation protocol into a scientific workflow for Machine Learning approaches, such as Bayesian Optimization, to drive intelligent scientific workflows (like SimStack^98,99^).

## Supporting Information Available

Computational details, simulated annealing, energetic analysis: equations, energy decomposition and competition, the lowest energy molecular adsorption configurations on Gr flakes, hybridization index, vibrational frequencies, and atomic coordinates (PDF)
