# Supplementary material for: Investigating Molecular Adsorption on Graphene-Supported Platinum Subnanoclusters: Insights from DFT + D3 Calculations
Source: ACS Omega. 2024 Sep 18;9(39):41067–83. doi: 10.1021/acsomega.4c07017 (PMC11447868; doi:10.1021/acsomega.4c07017)
Supplement: Supplementary file 1 — ao4c07017_si_001.pdf [file ao4c07017_si_001.pdf]

Supporting Information: Investigating Molecular Adsorption on Graphene-Supported Platinum Subnanoclusters: Insights from DFT+D3 Calculations

João Paulo Cerqueira Felix,[†] Gabriel Reynald da Silva,[‡] Glaucio R. Nagurniak,[¶] Alexandre C. Dias,[§] Renato P. Orenha,[‖] Celso R. C. Rêgo,[⊥] Renato L. T. Parreira,[‖] Diego Guedes-Sobrinho,[‡] and Maurício J. Piotrowski[*,#]

†*Institute of Physics "Armando Dias Tavares", Rio de Janeiro State University,* 20550-900*, Rio de Janeiro, RJ, Brazil*

‡*Chemistry Department, Federal University of Paraná,* 81531 − 980*, Curitiba, PR, Brazil*

¶*Department of Exact Sciences and Education, Federal University of Santa Catarina,* 89036-004*, Blumenau, SC, Brazil*

§*Institute of Physics and International Center of Physics, University of Brasília,* 70919 − 970*, Brasília, DF, Brazil*

‖*Núcleo de Pesquisas em Ciências Exatas e Tecnológicas, Universidade de Franca, Franca, SP, Brazil*

⊥*Institute of Nanotechnology Hermann-von-Helmholtz-Platz, Karlsruhe Institute of Technology,* 76021*, Karlsruhe, Germany*

#*Department of Physics, Federal University of Pelotas, PO Box* 354*,* 96010-900*, Pelotas, RS, Brazil*

E-mail: mauriciomjp@gmail.com

Contents

1 Computational Details **S3**

2 Simulated Annealing **S5**

3 Energetic Analysis: Equations **S6**

4 Energy Decomposition and Competition **S8**

5 The Lowest Energy Molecular Adsorption Configurations on Gr Flakes **S10**

6 Hybridization Index **S11**

7 Vibrational Frequencies **S12**

7.1 Pt Subnanoclusters S12

7.2 Pt Subnanoclusters Supported on Gr S12

7.3 Molecular Frequencies S13

8 Atomic Coordinates **S14**

1 Computational Details

Below, we provide the recommended ENMAX (maximum cutoff energy) values for constructing the projector augmented wave (PAW) potentials, along with the valence charge values for each chemical species involved in this study.

Table S1: ENMAX values of the projector augmented wave (PAW) potentials as provided in the POTCAR files for the species H, C, N, O, and Pt, with valences represented by Z_{val}, and maximum cuttof energies (ENMAX).

Element	Projector	Z_{val}	ENMAX (eV)
H	PAW PBE H 15Jun2001	01	250.000
C	PAW PBE C 08Apr2002	04	400.000
N	PAW PBE N 08Apr2002	05	400.000
O	PAW PBE O 08Apr2002	06	400.000
Pt	PAW Pt_pv_GW 23Mar2010	16	248.716

The electronic minimization algorithm employed in this study was the blocked Davidson iteration scheme (ALGO = Normal). To ensure convergence of the calculations, a minimum of six steps of electronic self-consistency (NELMIN = 6) was performed, with a maximum limit of 120 steps (NELM = 120). Additionally, eight non-self-consistent steps were conducted (NELMDL = −8). The normal precision calculation mode was selected (PREC = Normal), and spin polarization calculations were considered (ISPIN = 2). To evaluate augmentation charges, an additional support grid was utilized (ADDGRID = .TRUE.). Non-spherical contributions related to the density gradient within PAW spheres were included (LASPH = .TRUE.). Projection operators were evaluated in real space with fully automatic optimization (LREAL = AUTO). Lastly, the van der Waals (vdW) D3 correction was incorporated into the calculation (IVDW = 11).

We established a maximum limit of 200 ionic steps (NSW = 200) to determine how ions are updated and moved. The conjugate gradient algorithm (IBRION = 2) was utilized for ionic relaxation, with the scaling constant for step widths set to 0.50 (POTIM = 0.50). The stress tensor calculation was omitted to focus solely on force-criterion calculation. Ionic position relaxation (ISIF = 0) was performed to ensure a more stable structure. The Gaussian integration method (ISMEAR = 0) was employed for integration, and the smoothing width was set to

5 meV (SIGMA = 0.005) for fine-tuning the results. These settings were essential to ensure the precision and effectiveness of the calculations performed.

For the *Ab initio* molecular dynamics (AIMD) simulations, we adopted similar electronic minimization and ionic relaxation parameters. We opted for single grid precision (PREC = SINGLE), considering spin polarization calculations and vdW (D3) corrections. We set a maximum of 5000 ionic steps for ion updates (NSW = 5000). For the integration of motion equations, we used the Verlet algorithm (IBRION = 0), with a scaling constant for step widths set to 1.0 (POTIM = 1.0). The canonical ensemble was applied using the Nosé-Hoover thermostat (SMASS = 0), with the initial temperature at 300 K (TEBEG = 300), gradually reducing to 0 K (TEEND = 0). Additionally, we adopted a condition that simplifies Brillouin zone sampling (ISYM = 0).

2 Simulated Annealing

Below, we illustrate the temperature reduction (from 300 K to 0 K) achieved through the Simulated Annealing (SA) process in one of the AIMD simulations performed. SA is employed to explore the potential energy surface (PES), aiming to find the global minimum energy configuration. This method simulates the gradual cooling of a system, allowing it to escape local minima and reach the global energy minimum.

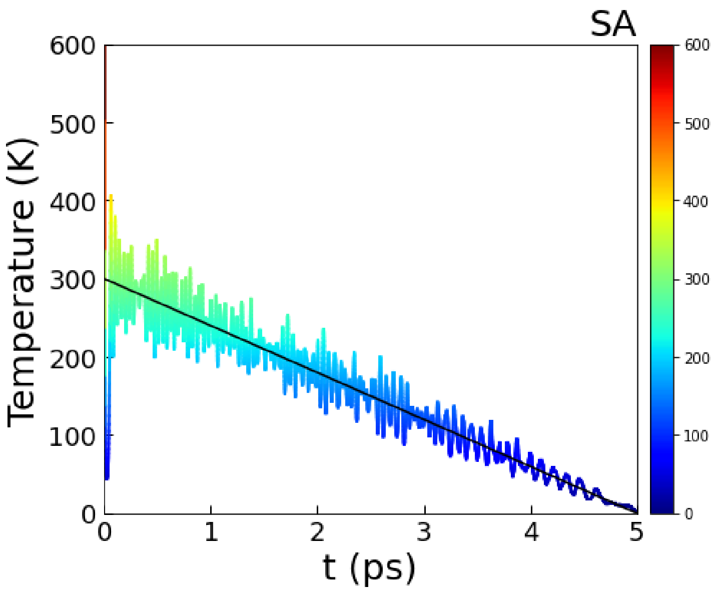

Figure S1: Simulated annealing simulation: temperature reduction from 300 K to 0 K over time (in ps).

3 Energetic Analysis: Equations

The binding energy of the adsorbed system, $E_{\text{b, ads}}$, can be expressed as:

$$E_{b,\text{ads}} = \frac{2E_b^{\text{mol}} + nE_b + \Delta E_{\text{int}} + n\Delta E_{\text{dis}}^{\text{clu}} + \Delta E_{\text{dis}}^{\text{mol}}}{2+n}\,, \tag{1}$$

where $2+n$ is the total number of atoms in the system. We can rewrite Equation (1) as:

$$\begin{aligned} E_{\text{b, ads}} = {} & \frac{2\left(\frac{E_{\text{tot}}^{\text{mol}} - E_{\text{tot}}^{\text{X}} - E_{\text{tot}}^{\text{Y}}}{2}\right) + n\left(\frac{E_{\text{tot}}^{\text{clu}} - nE_{\text{tot}}^{\text{Ag/Au}}}{n}\right) + \left(E_{\text{tot}}^{\text{mol/clu}} - E_{\text{tot}}^{\text{clu frozen}} - E_{\text{tot}}^{\text{mol frozen}}\right)}{2+n} + \\ & + \frac{n\left(\frac{E_{\text{tot}}^{\text{clu frozen}} - E_{\text{tot}}^{\text{clu}}}{n}\right) + \left(E_{\text{tot}}^{\text{mol frozen}} - E_{\text{tot}}^{\text{mol}}\right)}{2+n}\,, \end{aligned} \tag{2}$$

which can be simplified to:

$$\begin{aligned} E_{\text{b, ads}} = {} & \frac{E_{\text{tot}}^{\text{mol}} - E_{\text{tot}}^{\text{X}} - E_{\text{tot}}^{\text{Y}} + E_{\text{tot}}^{\text{clu}} - nE_{\text{tot}}^{\text{Ag/Au}} + E_{\text{tot}}^{\text{mol/clu}} - E_{\text{tot}}^{\text{clu frozen}} - E_{\text{tot}}^{\text{mol frozen}}}{2+n} + \\ & + \frac{E_{\text{tot}}^{\text{clu frozen}} - E_{\text{tot}}^{\text{clu}} + E_{\text{tot}}^{\text{mol frozen}} - E_{\text{tot}}^{\text{mol}}}{2+n}\,. \end{aligned} \tag{3}$$

Several factors cancel out in Equation (3), allowing us to rewrite the equation using the remaining terms as:

$$E_{\text{b, ads}} = \frac{E_{\text{tot}}^{\text{mol/clu}} - E_{\text{tot}}^{\text{X}} - E_{\text{tot}}^{\text{Y}} - nE_{\text{tot}}^{\text{Ag/Au}}}{2+n}\,. \tag{4}$$

The adsorption energy, E_{ads}, can be expressed as:

$$E_{\text{ads}} = \Delta E_{\text{int}} + n\Delta E_{\text{dis}}^{\text{clu}} + \Delta E_{\text{dis}}^{\text{mol}} \ , \tag{5}$$

which considers interaction energies and distortions. This Equation (5) simplifies to:

$$E_{\text{ads}} = E_{\text{tot}}^{\text{mol/clu}} - E_{\text{tot}}^{\text{clu frozen}} - E_{\text{tot}}^{\text{mol frozen}} + E_{\text{tot}}^{\text{clu frozen}} - E_{\text{tot}}^{\text{clu}} + E_{\text{tot}}^{\text{mol frozen}} - E_{\text{tot}}^{\text{mol}} \ . \tag{6}$$

Through appropriate simplifications, the terms reduce to:

$$E_{\text{ads}} = E_{\text{tot}}^{\text{mol/clu}} - E_{\text{tot}}^{\text{clu}} - E_{\text{tot}}^{\text{mol}} \ . \tag{7}$$

These two quantities can be related, transforming Equation (5) into Equation (1):

$$E_{\text{b, ads}} = \frac{2E_{\text{b}}^{\text{mol}} + nE_{\text{b}} + E_{\text{ads}}}{2+n} \ , \tag{8}$$

which provides an average over the total binding and adsorption energies.

4 Energy Decomposition and Competition

Below, we provide the nominal values of the binding energy terms for the composite systems (mol/clu), alongside the adsorption energy values for CO, NO, N_2, and O_2 molecules adsorbed on Pt_3 and Pt_6 nanoclusters. These terms account for penalties associated with distortions in the systems upon adsorption, as well as interaction terms.

$$(2+n)E_{\text{b, ads}} = 2E_{\text{b}}^{\text{mol}} + nE_{\text{b}} + E_{\text{ads}} \, , \tag{9}$$

where

$$E_{\text{ads}} = \Delta E_{\text{int}} + n\Delta E_{\text{dis}}^{\text{clu}} + \Delta E_{\text{dis}}^{\text{mol}} \, . \tag{10}$$

Table S2: Energy analysis of the lowest energy adsorbed systems (mol/clu): the terms $E_{\text{b}}^{\text{ads}}$ and E_{ads}.

	$2E_{\text{b}}^{mol}$ (eV)	nE_{b} (eV)	ΔE_{int} (eV)	$n\Delta E_{\text{dis}}^{\text{clu}}$ (eV)	$\Delta E_{\text{dis}}^{\text{mol}}$ (eV)
CO/Pt_3	-11.50	-7.03	-3.06	0.16	0.03
CO/Pt_6	-11.50	-19.11	-2.93	0.21	0.02
NO/Pt_3	-7.22	-7.03	-3.70	0.15	0.07
NO/Pt_6	-7.22	-19.11	-2.87	0.36	0.12
N_2/Pt_3	-10.40	-7.03	-1.61	0.04	0.03
N_2/Pt_6	-10.40	-19.11	-1.39	0.20	0.02
O_2/Pt_3	-6.08	-7.03	-1.83	0.02	0.07
O_2/Pt_6	-6.08	-19.11	-9.73	0.97	6.08

To properly account for pairwise interactions, the total interaction can be decomposed as follows:

$$\Delta E_{\mathrm{int}}^{\text{Gr-(mol/Pt}_n)} = \Delta E_{\mathrm{int}}^{\text{Gr-Pt}_n} + \Delta E_{\mathrm{int}}^{\text{mol/Pt}_n} + \Delta E_{\mathrm{int}}^{\text{Gr-mol}} \, . \tag{11}$$

While considering distortions induced in both the Gr–clu and mol/clu systems, it is essential to prioritize the interactions and distortions arising from the molecule's presence on the Gr surface. Given that the $\Delta E_{\mathrm{int}}^{\text{Gr-mol}}$ term typically contributes less significantly, we can effectively assess the predominant contribution to the Gr-(mol/clu) interaction by focusing on the remaining terms.

Table S3: Competing interactions in the adsorption of CO, NO, N_2, and O_2 on Gr-Pt_n systems.

		CO	NO	N_2	O_2
Gr-Pt_3	$\Delta E\mathrm{int}^{\text{Gr-Pt}_3}$ (eV)	-2.39	-2.39	-2.39	-2.39
	$\Delta E_{\mathrm{int}}^{\text{mol/Pt}_3}$ (eV)	-3.06	-3.70	-1.61	-1.82
Gr-Pt_6	$\Delta E_{\mathrm{int}}^{\text{Gr-Pt}_6}$ (eV)	-3.34	-3.34	-3.34	-3.34
	$\Delta E_{\mathrm{int}}^{\text{mol/Pt}_6}$ (eV)	-2.93	-2.87	-1.39	-9.73

Table S4: Properties of Gr-(mol/clu) and $\mathrm{Gr}^{\mathrm{flake}}$-(mol/clu).

sys. / prop.	E_{tot} (eV)	E_{Gr} (eV)	ΔE_{clu} (eV)	E_{mol} (eV)	E_{ads} (eV)	$d_{\text{mol-clu}}$ (Å)	m_{T} (μ_{B})	ν_{vib} (cm^{-1})
Gr-(CO/Pt_3)	-695.54	-666.92	-9.02	-14.80	-4.80	1.84	0.000	2011.22
$\mathrm{Gr}^{\mathrm{flake}}$-(CO/$Pt_3$)	-353.08	-324.52	-9.02	-14.80	-4.74	1.84	0.000	2011.01
Gr-(CO/Pt_6)	-709.48	-666.92	-23.08	-14.80	-4.68	1.94	0.000	1826.15
$\mathrm{Gr}^{\mathrm{flake}}$-(CO/$Pt_6$)	-485.28	-442.73	-23.08	-14.80	-4.67	1.95	0.000	1820.76
Gr-(NO/Pt_3)	-693.41	-666.92	-9.02	-12.32	-5.15	1.75	0.000	1811.18
$\mathrm{Gr}^{\mathrm{flake}}$-(NO/$Pt_3$)	-350.99	-324.52	-9.02	-12.32	-5.13	1.75	0.000	1819.63
Gr-(NO/Pt_6)	-707.07	-666.92	-23.08	-12.32	-4.75	1.96	1.000	1583.47
$\mathrm{Gr}^{\mathrm{flake}}$-(NO/$Pt_6$)	-482.85	-442.73	-23.08	-12.32	-4.72	1.97	1.000	1578.92
Gr-(N_2/Pt_3)	-696.07	-666.92	-9.02	-16.65	-3.48	1.90	0.000	2228.53
$\mathrm{Gr}^{\mathrm{flake}}$-($N_2$/$Pt_3$)	-353.59	-324.52	-9.02	-16.65	-3.40	1.90	0.000	2221.83
Gr-(N_2/Pt_6)	-709.78	-666.92	-23.08	-16.65	-3.13	1.95	2.000	2217.81
$\mathrm{Gr}^{\mathrm{flake}}$-($N_2$/$Pt_6$)	-485.48	-442.73	-23.08	-16.65	-3.02	1.95	2.000	2213.10
Gr-(O_2/Pt_3)	-689.17	-666.92	-9.02	-9.88	-3.35	1.87	0.000	1194.87
$\mathrm{Gr}^{\mathrm{flake}}$-($O_2$/$Pt_3$)	-346.81	-324.52	-9.02	-9.88	-3.39	1.88	0.000	1186.45
Gr-(O_2/Pt_6)	-705.60	-666.92	-23.08	-9.88	-5.72	1.77	0.000	788.00
$\mathrm{Gr}^{\mathrm{flake}}$-($O_2$/$Pt_6$)	-481.41	-442.73	-23.08	-9.88	-5.72	1.77	0.000	791.75

5 The Lowest Energy Molecular Adsorption Configurations on Gr Flakes

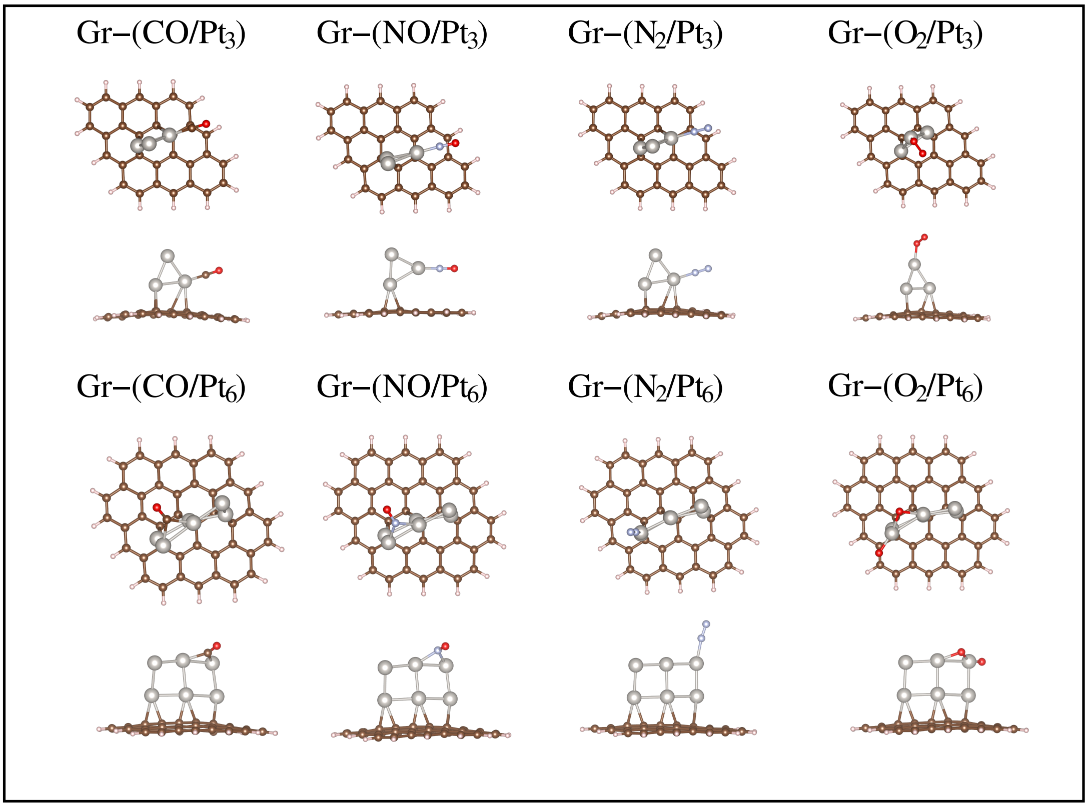

Figure S2: The lowest energy adsorbed configurations for the Gr-CO/$Pt_{3,6}^{flake}$, Gr-NO/$Pt_{3,6}^{flake}$, Gr-N_2/$Pt_{3,6}^{flake}$, and Gr-O_2/$Pt_{3,6}^{flake}$ systems, including top and side views for each case.

6 Hybridization Index

Below, we provided the *sd*, *sp*, and *pd* hybridization index values for the mol/clu systems.

Table S5: Hybridization indices computed for *sd*, *sp*, and *pd* cases, along with spin-polarized calculations providing values for both up and down spins, for the lowest energy adsorbed configurations of the mol/clu systems.

	Hybrid.	CO	NO	N_2	O_2
Pt_3	*sd* (*up*)	0.1035	0.1070	0.0921	0.0919
	sp (*up*)	0.0672	0.0947	0.1131	0.0727
	pd (*up*)	0.0204	0.0164	0.0183	0.0146
	sd (*down*)	0.1043	0.1027	0.0920	0.0919
	sp (*down*)	0.0668	0.0944	0.1131	0.0727
	pd (*down*)	0.0187	0.0148	0.0183	0.0146
Pt_6	*sd* (*up*)	0.1611	0.1269	0.1534	0.1254
	sp (*up*)	0.0713	0.0928	0.0995	0.0225
	pd (*up*)	0.0215	0.0204	0.0206	0.0275
	sd (*down*)	0.1585	0.1299	0.1562	0.1224
	sp (*down*)	0.0714	0.0930	0.1053	0.0247
	pd (*down*)	0.0208	0.0213	0.0200	0.0277

Table S6: Hybridization indices computed for *sd*, *sp*, and *pd* cases, along with spin-polarized calculations providing values for both up and down spins, for the lowest energy adsorbed configurations of the Gr-(mol/clu) systems.

	Hybrid.	CO	NO	N_2	O_2
Gr-Pt_3	*sd* (*up*)	0.6257	0.7369	0.6065	0.4623
	sp (*up*)	0.9798	1.3117	1.3749	1.0320
	pd (*up*)	0.1350	0.1202	0.1252	0.0978
	sd (*down*)	0.6257	0.7349	0.6065	0.4879
	sp (*down*)	0.9798	1.3115	1.3749	1.0272
	pd (*down*)	0.1350	0.1192	0.1252	0.0992
Gr-Pt_6	*sd* (*up*)	0.7041	0.6810	0.7484	0.3464
	sp (*up*)	1.1252	1.2130	1.3918	0.2948
	pd (*up*)	0.1330	0.1268	0.1316	0.0767
	sd (*down*)	0.7041	0.6706	0.7408	0.3640
	sp (*down*)	1.1252	1.2015	1.3653	0.3080
	pd (*down*)	0.1330	0.1241	0.1288	0.0781

7 Vibrational Frequencies

Below are the vibrational frequencies (in cm^{-1}) for the lowest-energy configurations of Ptn subnanoclusters (where $n = 2 - 7$ atoms). Following this, we present the vibrational frequencies of Ptn systems supported on graphene (Gr). Additionally, we provide the stretching frequencies of molecules adsorbed on Pt_n, both when supported and in the gas phase.

7.1 Pt Subnanoclusters

Pt_2

237.25;

Pt_3

233.14; 155.91; 153.44;

Pt_4

211.37; 196.88; 148.56; 137.37; 114.35; 24.27;

Pt_5

223.50; 167.22; 155.32; 155.13; 142.41; 93.32; 91.53; 69.17; 67.84;

Pt_6

237.99; 237.32; 196.21; 191.82; 171.22; 104.86; 90.30; 82.57; 81.44; 49.16; 42.77; 41.61;

Pt_7

253.18; 244.89; 178.78; 148.82; 146.00; 122.93; 120.29; 111.41; 104.90; 85.46; 72.19; 55.43; 49.81; 25.29; 20.33.

7.2 Pt Subnanoclusters Supported on Gr

Gr-Pt_3

220.12; 186.09; 144.38;

Gr-Pt_6

230.97; 215.63; 191.54; 183.69; 177.23; 144.65; 138.40; 101.77; 87.12; 74.72; 67.90; 66.21.

7.3 Molecular Frequencies

CO/Pt_3 & Gr-(CO/Pt_3)

2013.18 & 2011.22;

NO/Pt_3 & Gr-(NO/Pt_3)

1864.71 & 1811.18;

N_2/Pt_3 & Gr-(N_2/Pt_3)

2219.78 & 2228.53;

O_2/Pt_3 & Gr-(O_2/Pt_3)

1288.82 & 1194.87;

CO/Pt_6 & Gr-(CO/Pt_6)

2030.69 & 1826.15;

NO/Pt_6 & Gr-(NO/Pt_6)

1647.93 & 1583.47;

N_2/Pt_6 & Gr-(N_2/Pt_6)

2237.65 & 2217.81;

O_2/Pt_6 & Gr-(O_2/Pt_6)

810.70 & 788.00.

8 Atomic Coordinates

Pt

Pt_2

Pt -1.1613750317866334 -0.0532129935678682 0.0000000000000000

Pt 1.1613750317866334 0.0532129935678682 0.0000000000000000

Pt_3

Pt -1.2754402505183347 0.6591665528910529 0.0000000000000000

Pt 1.2086486628902193 0.7750705827055260 0.0000000000000000

Pt 0.0667915876281153 -1.4342371355965788 0.0000000000000000

Pt_4

Pt 1.2745300407703475 -0.1674855784561728 -0.0397883731341615

Pt -1.2564954708184022 -0.2739456011167398 0.0266890615224504

Pt -0.0666518035028609 0.2811828053839811 -2.1043060339183084

Pt 0.0486172335509174 0.1602483741889333 2.1174053455300168

Pt_5

Pt -1.8721153850361922 -1.3005303361734573 -0.0400273888681859

Pt -1.9599574273037437 1.1639915895460824 -0.0418649474075323

Pt 0.5062306880241874 1.2254232928246864 0.0118546023188095

Pt 0.5922350964944734 -1.1864101081484542 0.0126433276632412

Pt 2.7336070278212699 0.0975255619511444 0.0573944062936675

Pt_6

Pt -2.4193543382464462 -1.4303428001758043 0.0000000000000000

Pt 0.0165560634476147 -1.5389298290937123 0.0000000000000000

Pt 2.4497146063651005 -1.3795165657420370 0.0000000000000000

Pt -1.3419624273413291 0.7556537364366722 0.0000000000000000

Pt 1.3240374495794818 0.7829155209757435 0.0000000000000000

Pt -0.0289913538044182 2.8102199375991379 0.0000000000000000

Pt_7

Pt -0.8741543644101437 -2.0926881993560791 0.9474121315436932

Pt -0.9743371690896847 2.0714887405766564 0.8957508693492411

Pt 0.8471531413949815 2.1387187439332394 -0.9932416666534003

Pt 0.9494285985911883 -2.1197106100144003 -0.9401592620857997

Pt 0.1092384430084916 0.0034451992874889 0.0561318151663883

Pt -0.7473278311950740 0.0122036164982511 2.4043517151494740

Pt 0.6899991817002462 -0.0134574909251555 -2.3702456024695993

mol/clu

CO/Pt_3

Pt -2.7621577824825438 -0.3506535847811421 0.0435607770680928

Pt -0.3406037093299634 0.5175880747982884 0.0062306076390133

Pt -0.9786351657722943 -1.9743819262161217 -0.0014562634042719

C 1.4615980304613370 0.8342295796889303 -0.0173014758620234

O 2.6197986271234655 0.9732178565100522 -0.0310336454408127

NO/Pt_3

Pt -2.5871426478672639 -0.0735493179570721 0.0167816773489449

Pt -0.0246492553211848 -0.0184712920087424 -0.0039966003360359

Pt -1.4249315677587244 -2.1658050729609997 0.0239913952699684

N 1.5042718163156579 0.8379582115090649 -0.0168123502429616

O 2.5324516546315152 1.4198674714177422 -0.0199641220399105

N_2/Pt_3

Pt -2.7443817028156925 -0.3083540331600592 0.0343545063233108

Pt -0.3820483166017361 0.4582022992846380 0.0008243133602637

Pt -0.9590927326981600 -1.9816635004508303 0.0081435235377647

N 1.4856438804545249 0.8109785348187657 -0.0183356770541288

N 2.5998788716610655 1.0208366995074840 -0.0249866661672105

O_2/Pt_3

Pt 0.0292386218719933 -0.1162949387070995 0.4120609565022360

Pt 1.8801730081558556 -1.6208926607941923 -0.1674918859540053

Pt -0.4584089914269924 -2.4400321217403036 -0.2118323349056794

O -0.5690413335288866 1.6081223731152079 0.3769254717562767

O -0.8819613050719610 2.5690973481263857 -0.4096622073988243

CO/Pt_6

Pt -0.7804856933410420 -2.0268398404712284 -0.0000003749999937

Pt -1.1336852714521566 2.2819040348946107 -0.0000003749999937

Pt -3.2328249533863858 -2.0973489630814424 -0.0000003749999937

Pt 0.5547773089664343 0.3617924287222714 -0.0000003749999937

Pt -2.1689525485506156 0.1134658363727965 -0.0000003749999937

Pt 1.6219360500112820 -1.9616431407583761 -0.0000003749999937

C 2.0803785293395833 1.3484954749733973 -0.0000003749999937

O 3.0588565784128967 1.9801741693479755 0.0000026249999703

NO/Pt_6

Pt 3.1051073753812695 -1.2944978462543038 -1.6811890028215171

Pt -1.4295224347356346 -1.7089918988348831 0.4634256959721093

Pt 0.8450351066874831 2.7924073958602769 -0.1041235648552430

Pt 1.9317706018643186 0.7541250560297819 -0.8939651909927555

Pt 0.8963174568919019 -1.5994473598558212 -0.6463890182914884

Pt -0.3603850923110361 0.6389275018647869 0.2492036660879666

N -1.9789210920180942 0.0870149036918360 1.0192297130210299

O -3.0094019217602028 0.3304622474983283 1.5938077018799000

N_2/Pt_6

Pt 3.1612648734600945 -1.0608311572797273 -1.9208909134794556

Pt -1.2140513625876146 -2.2307956489156862 -0.1958170330852376

Pt 0.7394347429708059 2.3905404842702254 0.4673650579497224

Pt 1.9363610796919684 0.6842313272244418 -0.7160666740982311

Pt 0.9488293610976317 -1.6511182249055256 -1.0516106489044379

Pt -0.5225118872897117 0.1745595516444958 0.3159457551815859

N -2.0619118825824856 0.6913140411749765 1.2642059294653230

N -2.9874149247606852 1.0020996267868014 1.8368685269707292

O_2/Pt_6

Pt 2.2665861416454689 -0.4689951734491604 -1.4176911159956784

Pt -2.2482384211463868 -2.3304619402033335 -0.4025661632873199

Pt 1.9263178201532440 1.9954258491710863 -1.3823239282683506

Pt 0.0888731375904825 0.8707711061151784 -0.0321726034939580

Pt -0.0245177655662907 -1.3933183772958966 -1.0142567613269584

Pt -1.2535146380815743 -0.8188706177127383 1.2480741063115417

O 0.8689520662271200 2.6239504302741850 0.0706227060605027

O -1.6244583408220628 -0.4785012768993244 2.9303137600002298

Gr-clu

$Gr-Pt_3$

C 0.7069869864437424 0.2976929557035897 0.0836321806136073

C 3.1943331952628027 0.2976929557035897 0.0836321806136073

C 5.6736523938640699 0.3122077756192780 -0.0952152215189059

C -6.6822095992247679 0.3168461804021163 -0.1582675332480292

C -4.2175819801976164 0.3168461804021163 -0.1582675332480292

C -1.7555720593863313 0.3122077756192780 -0.0952152215189059

C 0.7143257954617495 2.4678540480005875 -0.0342164669052814

C 3.2099428415703821 2.4788329289823583 0.0049622259187494

C 5.6928794502983591 2.4678540480005875 -0.0342164669052814

C -6.6799121569537530 2.4586896176785160 -0.1337974387525414

C -4.2137949668817765 2.4578356088128963 -0.1611663289136160

C -1.7466906523129619 2.4586896176785160 -0.1337974387525414

C 0.7229995607396971 4.6002430873867990 -0.1322883709288032

C 3.1941858598070274 4.5989241960291736 -0.1024863105323526

C 5.6737683508142256 4.5989241960291736 -0.1024863105323526

C -6.6767503079578265 4.6002430873867990 -0.1322883709288032

C -4.2103935697907300 4.5996029956577473 -0.1611812582262520

C -1.7440962916916529 4.5996029956577473 -0.1611812582262520

C 0.7268380858227017 -6.0994460888944717 -0.1678141338575578

C 3.1950514947679194 -6.1024813470225201 -0.1528786978819259

C 5.6663119453251634 -6.1039010594911653 -0.1469933018578846

C -6.6840170261658463 -6.1024813470225201 -0.1528786978819259

C -4.2122968030258860 -6.0994460888944717 -0.1678141338575578

C -1.7416839555797354 -6.0976345317209049 -0.1752652475210876

C 0.7288113117957709 -3.9605142485070122 -0.1675949921719457

C 3.1974247635569588 -3.9653689830100634 -0.1667629214477717

C 5.6667047815068949 -3.9652587393015484 -0.1675742356058159

C -6.6878163347244399 -3.9652587393015484 -0.1675742356058159

C -4.2186644976442205 -3.9653689830100634 -0.1667629214477717

C -1.7444452813386526 -3.9605142485070122 -0.1675949921719457

C 0.7241035406496996 -1.8267653075979045 -0.1237152148407041

C 3.2000585921969060 -1.8292248192391458 -0.1204487295003887

C 5.6685155807744794 -1.8262748551613868 -0.1553742185784408

C -6.6868021173464145 -1.8255384232582390 -0.1693668041268932

C -4.2197422913824569 -1.8262748551613868 -0.1553742185784408

C -1.7546925103136184 -1.8292248192391458 -0.1204487295003887

C 1.5499165725785113 1.7584577658731693 0.0990974619300875

C 4.0381515218820647 1.7584577658731693 0.0990974619300875

C 6.5020263684538202 1.7474328506349224 -0.0879062162487418

C -5.8588840679218546 1.7430888339949622 -0.1548419434925101

C -3.3940233515317244 1.7430888339949622 -0.1548419434925101

C -0.9266888671544660 1.7474328506349224 -0.0879062162487418

C 1.5461879464701012 3.8878645768883686 -0.0897396284473349

C 4.0221380086805505 3.8855962240743072 -0.0799713778040694

C 6.5007052215066077 3.8878645768883686 -0.0897396284473349

C -5.8570470382335778 3.8859026655041724 -0.1418885372686933

C -3.3896620867614198 3.8852756293645072 -0.1625736303952614

C -0.9215530963213112 3.8859026655041724 -0.1418885372686933

C 1.5493672365635396 6.0245950012111482 -0.1536250988113324

C 4.0177380226153074 6.0196701949172038 -0.1363796766955225

C 6.4907523578276711 6.0196701949172038 -0.1363796766955225

C -5.8584190620414338 6.0245950012111482 -0.1536250988113324

C -3.3889785507772725 6.0247221175488175 -0.1696981184094160

C -0.9199264933969671 6.0247221175488175 -0.1696981184094160

C 1.5516236952297051 -4.6755925547479489 -0.1694719369565654

C 4.0199424559418739 -4.6786432961259479 -0.1654816142827116

C 6.4882506441052792 -4.6802614725016713 -0.1638707142349833

C -5.8647994233989529 -4.6786432961259479 -0.1654816142827116

C -3.3929599699751893 -4.6755925547479489 -0.1694719369565654

C -0.9197734542231952 -4.6740437841016895 -0.1741236708200429

C 1.5523997992266070 -2.5398255917026922 -0.1420760057612149

C 4.0238032027622408 -2.5410477818580626 -0.1530125626124548

C 6.4909288914788830 -2.5400335606265494 -0.1682272587931575

C -5.8663331635632012 -2.5400335606265494 -0.1682272587931575

C -3.4003794790485617 -2.5410477818580626 -0.1530125626124548

C -0.9275638118824885 -2.5398255917026922 -0.1420760057612149

C 1.5356098029900567 -0.4211952305690216 -0.0274827657009613

C 4.0319328749273415 -0.4094778491425730 -0.0544183549081811

C 6.4937272074795542 -0.3989305237248653 -0.1429813336146886

C -5.8625969039277690 -0.3979731177588217 -0.1667501836497216

C -3.3967996501195605 -0.3989305237248653 -0.1429813336146886

C -0.9471842023055865 -0.4094778491425730 -0.0544183549081811

Pt 2.3107294519072186 0.9213080820255097 4.4126700781847656

Pt 1.1264588554084876 0.9875576028842286 2.2297426407956902

Pt 3.5714504058090411 0.9875576028842286 2.2297426407956902

Gr-Pt_6

C 0.9107296818385784 0.2073446085117983 -0.2025121199103399

C 0.9540112127270959 2.3863542570465537 -0.0270549927664643

C 0.9214802213535220 4.5097621407229127 -0.2072753170019439

C 0.9167172021325021 -6.1813704052245484 -0.3061426851836995

C 0.9163211593286356 -4.0468476295356677 -0.3501448341027853

C 0.9138905854601598 -1.9145059337773898 -0.3278534497061329

C 3.3866488366908367 0.2279762977706392 -0.2760100585144549

C 3.4113467642079023 2.3748488726277426 -0.2024850953466419

C 3.3916124472189466 4.5103078295712020 -0.3002335482545426

C 3.3851245291075429 -6.1790366486437911 -0.3536585488717936

C 3.3813242645084323 -4.0453381443349041 -0.3585863664400506

C 3.3814206423387478 -1.9107232584769598 -0.3350725722890502

C 5.8483736894086942 0.2289456625145672 -0.3110814759337686

C 5.8575841673691560 2.3661427604453387 -0.3089016729794611

C 5.8584323860482215 4.5055572107525403 -0.3436101678859167

C 5.8535184771419804 -6.1782981282469365 -0.3853989435334615

C 5.8479478416983195 -4.0469259124483763 -0.3739976969991119

C 5.8475856406888873 -1.9092972687210601 -0.3302031482979331

C -6.4944425236717134 0.2251867502074338 -0.2413035974349818

C -6.4846468813403000 2.3674036632227402 -0.3006231859141515

C -6.4820058906457456 4.5030533493966232 -0.3480052978674362

C -6.4820361971657841 -6.1810068365076853 -0.3920705226014469

C -6.4820160528312032 -4.0428898192351710 -0.3785582863998602

C -6.4938338915541838 -1.9145370393822558 -0.2958259554580493

C -4.0340837004988792 0.2070746708826015 -0.0421943437714809

C -4.0350339753819036 2.3754832934004195 -0.1341478855604485

C -4.0175144537918559 4.5011613616828043 -0.2835464752014492

C -4.0154590597382827 -6.1828192231958941 -0.3688692656899892

C -4.0175614677139571 -4.0482071799606016 -0.3889177010434324

C -4.0173018582810691 -1.9151923616151612 -0.3154286167563800

C -1.5620115883983816 0.2042086162041548 -0.1827512425002578

C -1.5360108829002277 2.3823409037400847 0.0997373034462754

C -1.5452209122540292 4.5045176184807385 -0.1748806168490464

C -1.5507311000208475 -6.1830059111522973 -0.3209429545488867

C -1.5511082067184345 -4.0498653997678851 -0.3682290633221132

C -1.5511818129718042 -1.9157389839266461 -0.3348930628270228

C 1.7577076181859059 1.6450099974391339 -0.0729547174791882

C 1.7577410296420624 3.8087852628636085 -0.1957268940513330

C 1.7413377447527463 5.9294011926477328 -0.3007107975406971

C 1.7389174647487931 -4.7570555469609772 -0.3447081856049099

C 1.7377460825309115 -2.6243444395442741 -0.3440388870229452

C 1.7354926894372902 -0.4904950230821932 -0.2733314592647869

C 4.2171208805191824 1.6528318673231652 -0.2614678778425628

C 4.2160010375221493 3.7949579687447450 -0.2835047158248756

C 4.2102820839047581 5.9302334172446454 -0.3599930709275583

C 4.2047877592444802 -4.7573819070817622 -0.3703506972097816

C 4.2019957987914509 -2.6213944382642809 -0.3475296155076766

C 4.2043456212861496 -0.4831722095617987 -0.3125120855702530

C 6.6730334527282231 1.6524112092355967 -0.3138536375214223

C 6.6781674233127788 3.7916347751645141 -0.3348857573386166

C 6.6768588437658032 5.9275144882257234 -0.3812601724545690

C 6.6724707738316198 -4.7564159933522445 -0.3854440680831690

C 6.6674109261957977 -2.6229681049743228 -0.3411840532009141

C 6.6671143313532468 -0.4862141861862135 -0.3015981331173681

C -5.6679438570264189 1.6581476241611988 -0.2374031265649403

C -5.6607493925554850 3.7905260472938735 -0.3125812093877798

C -5.6610338834919780 5.9258082130496650 -0.3740719946396549

C -5.6597659385168306 -4.7566531011775393 -0.3940571316507757

C -5.6633198331779546 -2.6241736499864112 -0.3311209179326315

C -5.6879369162991766 -0.4978079011768886 -0.2003428293716603

C -3.2293890278551021 1.6510044715543835 0.0731738666615733

C -3.2004997269893445 3.7906320129107360 -0.1907337456495917

C -3.1939314987663439 5.9259917403245321 -0.3263329393392542

C -3.1941769236746294 -4.7602063722709289 -0.3828100113830324

C -3.1947995035332766 -2.6256034911026700 -0.3595683423241383

C -3.1906308342946144 -0.5001240184178499 -0.2078109256900742

C -0.7354441908970779 1.6367314408241089 0.0169169244067238

C -0.7145292171807869 3.8097953766276813 -0.0902808074975194

C -0.7268201319879983 5.9247578702891399 -0.2715732474180435

C -0.7290959267996344 -4.7606722845061693 -0.3522979285258394

C -0.7276165510098593 -2.6256767343256335 -0.3495763803152858

C -0.7292313963177222 -0.4956716803646302 -0.2590978359819633

Pt -3.9074565814779239 0.7698608117814416 4.5985288827180106

Pt -1.1437408160217357 1.5427024963960072 4.6060656122978330

Pt 1.6100146848394470 2.2920953252989431 4.5786059934432828

Pt -1.0651665779002792 1.7886655183747013 2.1528627608780138

Pt 1.6332130315485713 2.1239369027500192 2.0988950503928070

Pt -3.9503498457566297 0.7405312687839549 2.1113385727542120

Gr-(mol/clu)

Gr-(CO/Pt_3)

C 0.5613736220382926 0.2721878365145542 0.0270595837926759

C 3.0514741805420620 0.2757656054459385 -0.0236423490391378

C 5.5246326732836000 0.2874282720638437 -0.2207064336305962

C -6.8308878552576546 0.2899619101672659 -0.2696694730800235

C -4.3646938663531714 0.2893852346029480 -0.2615569149751575

C -1.9009802632207951 0.2849696234969983 -0.1912069681652291

C 0.5674838136093969 2.4410865874162795 -0.1264493062370580

C 3.0703993657581528 2.4493293438352381 -0.0686533918981951

C 5.5400972934453581 2.4382601810228284 -0.1563602239611530

C -6.8291059223555948 2.4306232179827205 -0.2518586011978030

C -4.3614250499386333 2.4302366146664429 -0.2681279094800129

C -1.8934126121545738 2.4313075613495112 -0.2370678350125495

C 0.5767659039836799 4.5726444194258367 -0.2253396091224840

C 3.0495163542524111 4.5714001469648711 -0.1842137891529259

C 5.5277175584624594 4.5722364268410214 -0.1925661609243896

C -6.8250969178570600 4.5721216595023293 -0.2351053127385150

C -4.3584429920904011 4.5716524209199267 -0.2578474402448894

C -1.8912379752116468 4.5717162365121116 -0.2539250043394627

C 0.5802475786742516 -6.1268869743384000 -0.2492994918822777

C 3.0489605596229552 -6.1294735367972990 -0.2370757296896340

C 5.5208736124168141 -6.1303576199212699 -0.2314277834026868

C -6.8302038912235847 -6.1288498863484859 -0.2410580743148838

C -4.3588293505802138 -6.1265313080857000 -0.2505925481747564

C -1.8887180669458363 -6.1257030895451772 -0.2525768416458760

C 0.5816338171987541 -3.9889253614072095 -0.2336803016686435

C 3.0511253029925900 -3.9913774313725554 -0.2483218902970297

C 5.5204491878569097 -3.9911913763479241 -0.2526851456390737

C -6.8339175951774669 -3.9916320866160211 -0.2519818147201729

C -4.3643032937457997 -3.9917866443796144 -0.2440880209761662

C -1.8896579639103397 -3.9867916181963383 -0.2299175711692723

C 0.5800536982314499 -1.8545462511060684 -0.1692476986603424

C 3.0535059669517466 -1.8538305663056658 -0.2056670162652701

C 5.5214317848663068 -1.8519980528742490 -0.2525806133260069

C -6.8335990451625150 -1.8518560481427233 -0.2632615408930121

C -4.3660213610563181 -1.8527922766295228 -0.2430196390376089

C -1.9001378815919825 -1.8553262858973749 -0.1897491915521616

C 1.4044701593954318 1.7321435638936187 0.0315042433598123

C 3.8907879480902121 1.7295658841231187 -0.0242890180233619

C 6.3503447976799539 1.7179560321233645 -0.2178445913070064

C -6.0078673668183917 1.7155063853277372 -0.2685569485524031

C -3.5409523600690593 1.7157821411512479 -0.2608771808446804

C -1.0723451458869908 1.7200244337836139 -0.1887837889640878

C 1.3995922898411788 3.8600621287987114 -0.1809161126176040

C 3.8795418575738161 3.8597552997940685 -0.1581264187213254

C 6.3518815020070409 3.8592389031977099 -0.1964737017725060

C -6.0055444469418457 3.8575008361445722 -0.2485047364783064

C -3.5373369277481017 3.8572027604961416 -0.2616608234754452

C -1.0687257643156869 3.8578803429692652 -0.2392831444623873

C 1.4029616034122068 5.9970545525426395 -0.2400455151752094

C 3.8716769093569052 5.9920578054971472 -0.2197789950421107

C 6.3455352292229446 5.9944071189005825 -0.2247298708594307

C -6.0054036991342574 5.9969935140633677 -0.2446333507290017

C -3.5362014704722293 5.9967062824020037 -0.2546452826335486

C -1.0668273874299876 5.9969940439090514 -0.2528050114043516

C 1.4048152178092348 -4.7030011708973012 -0.2458281195377872

C 3.8734240169620087 -4.7052100088672795 -0.2488040104398657

C 6.3422511036340374 -4.7061638689447403 -0.2484108786812023

C -6.0106478748776349 -4.7050339859502337 -0.2486013077901834

C -3.5386698290377581 -4.7023293584347954 -0.2429359869144054

C -1.0659654296089691 -4.7014966123128286 -0.2398306787274915

C 1.4058770761467656 -2.5667694237381569 -0.2035447409936495

C 3.8768037306655661 -2.5666131540945489 -0.2415131467487690

C 6.3437239014431555 -2.5662361912396436 -0.2592859454151348

C -6.0126520769638336 -2.5664562189454432 -0.2560227010703144

C -3.5457815785721785 -2.5675652263570203 -0.2309989228603175

C -1.0714787250841518 -2.5662870820387558 -0.1954719713778239

C 1.3989527552110310 -0.4446443460020815 -0.0747738647802549

C 3.8826917362458637 -0.4332015157372133 -0.1614251045481350

C 6.3455309192935232 -0.4252015145896557 -0.2553051384015053

C -6.0100036543523849 -0.4249507507458450 -0.2693453201028788

C -3.5434269608752871 -0.4261125923928999 -0.2396163743070190

C -1.0940317274386562 -0.4365085096969095 -0.1327170961800945

Pt 1.8579065249372242 0.9631182982009880 4.4131982193883168

Pt 0.9299335207154709 0.9905158212149248 2.1025023945671393

Pt 3.3911478229267580 0.9652964659461913 2.4196030710646976

C 5.1373294234096090 0.9576056670733184 2.9868119115410483

O 6.2396120092937295 0.9579563650130982 3.3662340127402413

Gr-(NO/Pt_3)

C 0.5976484293679425 0.2784110800571282 -0.0858528459529015

C 3.0753205571903974 0.2872416394105821 -0.2500623625434137

C 5.5436847877340041 0.2906169228419504 -0.2678246268203370

C -6.8115709308006878 0.2903856904889812 -0.2526720963335247

C -4.3431685120397345 0.2903021165239013 -0.2422562382921232

C -1.8789883349618943 0.2867094122972214 -0.2075639661688307

C 0.5923653856035402 2.4385143078017979 -0.1622839593522745

C 3.0934790091155131 2.4391852392854751 -0.1901842302135588

C 5.5441547602830452 2.4306547385157806 -0.2622527383098259

C -6.8107565582317697 2.4300587499445943 -0.2559147750095434

C -4.3414046172261358 2.4300960762126298 -0.2456739364611398

C -1.8718683624372288 2.4306255982512450 -0.2310564092812086

C 0.5985819623159658 4.5714281726711050 -0.2160900353354158

C 3.0719234014224410 4.5693001935231479 -0.2050372239768770

C 5.5474931537497492 4.5712945626080543 -0.2332294927622200

C -6.8089060954250691 4.5704162849436365 -0.2428927518732333

C -4.3400530562612385 4.5698663677056910 -0.2385156868825611

C -1.8700579158080624 4.5705515005090493 -0.2318221311201967

C 0.6003593188097289 -6.1282487719853140 -0.2272642982643820

C 3.0704639611158475 -6.1293215638573759 -0.2226020343251367

C 5.5426302188872141 -6.1293585069561027 -0.2264467803259400

C -6.8097780570210347 -6.1284645302591043 -0.2317144622059502

C -4.3387858583669310 -6.1269792458144821 -0.2292494711226869

C -1.8688079052841333 -6.1268001172001902 -0.2278528815908967

C 0.6023308196567276 -3.9898116595096282 -0.2296552975534958

C 3.0718105476440707 -3.9897123538027146 -0.2342066168699191

C 5.5413789948689169 -3.9902211033965655 -0.2325538648196535

C -6.8118238156664592 -3.9901834174869775 -0.2294915450437074

C -4.3422436491952574 -3.9903359374671457 -0.2217227548248424

C -1.8677924152501122 -3.9864148960627128 -0.2174078019045975

C 0.6049509377611662 -1.8520874830989085 -0.2145443880293474

C 3.0728718376204514 -1.8509891880968254 -0.2468047964202427

C 5.5422572147725448 -1.8497618123950401 -0.2480718504152897

C -6.8123220250613370 -1.8501180259967436 -0.2397645418825292

C -4.3440605112029012 -1.8520905068759266 -0.2234620536671130

C -1.8759441928558225 -1.8534760137146771 -0.1948788767962224

C 1.4338455390228271 1.7272577897554271 -0.0833772083568611

C 3.9021354212347186 1.7192652804716246 -0.2482009093210706

C 6.3668601399597877 1.7165427944007856 -0.2670467583272718

C -5.9880819137493049 1.7165615017043141 -0.2523616549950987

C -3.5200144595323231 1.7160770007186610 -0.2426282933332295

C -1.0518797462182130 1.7194079204365131 -0.2083797419336246

C 1.4220528936244952 3.8591808082961467 -0.1954410466511902

C 3.9016987115044630 3.8580688638012361 -0.2123598534827451

C 6.3685292525022925 3.8576469258350645 -0.2453947213916372

C -5.9866184554269237 3.8565414431281244 -0.2464561695886562

C -3.5172147244760330 3.8568628463805013 -0.2391498171698565

C -1.0471703214105696 3.8582028520551308 -0.2253541634872143

C 1.4236144407794056 5.9965206307348033 -0.2231367890066043

C 3.8936071648846040 5.9927853219848934 -0.2187426667126129

C 6.3677124311368596 5.9962486552041270 -0.2314636358320694

C -5.9861400735613799 5.9964631338789900 -0.2345766685601500

C -3.5159731762197088 5.9967660943611119 -0.2307039563390703

C -1.0457935444845496 5.9965892992580550 -0.2290428393867234

C 1.4253937721025176 -4.7023946178602323 -0.2305713218834455

C 3.8947070922762927 -4.7037032795228688 -0.2298706565360789

C 6.3642338848067075 -4.7038756034896316 -0.2298131551857079

C -5.9883803084792575 -4.7027143841512986 -0.2276367378021025

C -3.5165999964127317 -4.7014719281086403 -0.2212624039648325

C -1.0445008429228446 -4.7013360951500633 -0.2242119754480818

C 1.4275432781661292 -2.5648857354484957 -0.2337773808783243

C 3.8957404317737323 -2.5637025533517788 -0.2435820464163978

C 6.3648646873004848 -2.5632976117655111 -0.2401069228473158

C -5.9894769256096021 -2.5640759592454336 -0.2312404854899626

C -3.5217704691147556 -2.5653547155346152 -0.2143043584715194

C -1.0459752076195992 -2.5631917272504761 -0.2052537963094263

C 1.4352359989507102 -0.4333403043512236 -0.1937377527672890

C 3.8962662752054671 -0.4238044393966787 -0.2642733405969633

C 6.3651740880559471 -0.4231275507496468 -0.2572969262281166

C -5.9889436365134436 -0.4233130558470437 -0.2458258212713158

C -3.5202225637062168 -0.4244441070190286 -0.2293798074466640

C -1.0651875262679180 -0.4327552942003567 -0.1622674233406300

Pt 0.9495988492708571 0.9674198949993409 4.4489753624164852

Pt 0.9138004012420344 0.9908386482729750 1.9962472863340714

Pt 3.1642182751027539 0.9576750951708082 3.3199670742873639

N 4.9079750759649459 0.9433478520927006 3.2235319472293593

O 6.0897633020338331 0.9332347898860736 3.2483873592439529

Gr-(N_2/Pt_3)

C 0.5604981922413268 0.2726516491773570 0.0221863797215303

C 3.0496847600413535 0.2761935091758057 -0.0178363044331267

C 5.5229367307461601 0.2877838507577337 -0.2120095944298868

C -6.8321866616776372 0.2905509796077848 -0.2618362885958057

C -4.3658347564982041 0.2900973013666501 -0.2551664638756748

C -1.9020174024025822 0.2854095167640862 -0.1886666615803083

C 0.5667363159472734 2.4417264155105221 -0.1270819092531159

C 3.0686515846254157 2.4497525202969905 -0.0700668615316449

C 5.5381040515297961 2.4383439219871912 -0.1480223590508309

C -6.8304468769149311 2.4312712284698064 -0.2436885319344899

C -4.3626432219193667 2.4307963872679386 -0.2607387968010357

C -1.8946745759977546 2.4319765786892118 -0.2321120669337251

C 0.5756657733021724 4.5732500977358592 -0.2226522327563174

C 3.0484145072261875 4.5720633772646329 -0.1838194885286324

C 5.5264159678985312 4.5727133197340910 -0.1887033200043486

C -6.8265030299902447 4.5726401306008588 -0.2288460929024794

C -4.3597806381373134 4.5721144702563015 -0.2518023547182633

C -1.8924839228976023 4.5722810595375689 -0.2495025260936306

C 0.5790322834480506 -6.1263865639143580 -0.2464618879543217

C 3.0479535711608055 -6.1287808336491807 -0.2344187531717701

C 5.5196676557937963 -6.1297358325676390 -0.2283982561384565

C -6.8314520903956799 -6.1283467713679753 -0.2372945335196519

C -4.3601814659122216 -6.1260499278869531 -0.2474487204370188

C -1.8899570972415161 -6.1252501376918005 -0.2499715469710289

C 0.5802977228661446 -3.9884587320933331 -0.2315778046528969

C 3.0498700205101024 -3.9909633961751370 -0.2446795919016882

C 5.5192965254727220 -3.9905586629854959 -0.2488800263609310

C -6.8350728146412427 -3.9909272320188327 -0.2491725988616977

C -4.3655142445788391 -3.9912713184012913 -0.2430237173391614

C -1.8910744699246695 -3.9864854350683645 -0.2300347270289294

C 0.5785456311635402 -1.8540134266908765 -0.1710817031545098

C 3.0519905494881190 -1.8535190338963998 -0.1996002245225714

C 5.5202366233781532 -1.8513107046396780 -0.2467095000269754

C -6.8347073670257457 -1.8511943131213062 -0.2580877302997528

C -4.3671566713373711 -1.8521395802631400 -0.2401964602381970

C -1.9012514991801410 -1.8548829408978991 -0.1906982981729310

C 1.4035729712701706 1.7324798424210082 0.0269967277957726

C 3.8888698483290955 1.7296874046650954 -0.0174714318256584

C 6.3488362668345246 1.7186718176838651 -0.2085470196410260

C -6.0091511040859391 1.7161119792970121 -0.2604502907555855

C -3.5421693865515955 1.7164804990060647 -0.2541131056227961

C -1.0732902424172437 1.7207139639407556 -0.1857149704359369

C 1.3986430260469049 3.8607344100654233 -0.1802246762227231

C 3.8780127819093426 3.8600128530518321 -0.1577246485819881

C 6.3504787176243518 3.8598085129890540 -0.1893015656387558

C -6.0069899437678078 3.8580044711112889 -0.2415572463413067

C -3.5384537175287458 3.8578143607356283 -0.2554732852026103

C -1.0699020075293060 3.8585004951698751 -0.2351417096249619

C 1.4019942694744643 5.9977534904067307 -0.2372889970920937

C 3.8705559205341995 5.9926974793049110 -0.2176205348356657

C 6.3442734333923312 5.9948839236185796 -0.2208321498693735

C -6.0066030224802898 5.9975014157435078 -0.2403652869814881

C -3.5374961804793403 5.9971941456474438 -0.2505740476161549

C -1.0680575125600695 5.9974407485993071 -0.2497204206175692

C 1.4034189096734515 -4.7025574662852119 -0.2428581432394434

C 3.8722123731736016 -4.7047020984261154 -0.2454430255430591

C 6.3411918030507675 -4.7055303258579242 -0.2449995852658589

C -6.0119181068967977 -4.7044817557091188 -0.2460898449526248

C -3.5401029448049912 -4.7019961205396310 -0.2417677654075714

C -1.0672464292748174 -4.7010644236000338 -0.2387519740168358

C 1.4045082025980147 -2.5663757838124401 -0.2014699043173298

C 3.8755383503790828 -2.5660856237878629 -0.2361464763307453

C 6.3425844439783035 -2.5655529174050766 -0.2541834857838285

C -6.0138328979595634 -2.5657348842278611 -0.2526617746140314

C -3.5469190640191455 -2.5669598748372007 -0.2299564718084781

C -1.0730252449631204 -2.5660586825600014 -0.1973230996090614

C 1.3970491417953763 -0.4443870945910398 -0.0780010301725973

C 3.8812301812021612 -0.4323781125800030 -0.1542846601420820

C 6.3441493589896698 -0.4246566255269029 -0.2479056781286442

C -6.0110107192186852 -0.4241365696261798 -0.2626511787216561

C -3.5446431481620047 -0.4254868814845310 -0.2354430632408135

C -1.0949785332745936 -0.4361250336810922 -0.1343061611811667

Pt 1.8573985471822727 0.9565191518451810 4.4146249874130259

Pt 0.9362994328378953 0.9895478068866366 2.1063304850073301

Pt 3.3706821696135467 0.9623815509202949 2.4092378198772355

N 6.2876848178047879 0.9409810445530979 3.1974376893016867

N 5.1955455781131032 0.9470074360048866 2.9098385544406291

$Gr-(O_2/Pt_3)$

C 0.6505664811849856 0.2740112866406630 -0.1269988080632896

C 3.1326397514768987 0.2740194147993948 -0.1253237642287139

C 5.6124062608206833 0.2864452858904230 -0.2746376706029388

C -6.7433875467564022 0.2899943787527572 -0.3274180659517452

C -4.2777997232753204 0.2899714317234752 -0.3280605602365494

C -1.8146029902384866 0.2862113423693176 -0.2768741173513654

C 0.6517688158716242 2.4404194691557137 -0.2036818817776549

C 3.1502043021641812 2.4533012013215956 -0.1743517467191236

C 5.6335643337321644 2.4405035134233746 -0.2002471734780933

C -6.7392899118266572 2.4316326683171656 -0.2992827200795620

C -4.2742407106691820 2.4312126494857846 -0.3283039835102226

C -1.8087314688600555 2.4317234920817139 -0.3010695706391182

C 0.6628108890897879 4.5741545739396017 -0.2935902473462733

C 3.1332357462238134 4.5726355870199580 -0.2713417085060037

C 5.6144238786526524 4.5726999436433111 -0.2710914096951633

C -6.7368113190498491 4.5740323591570657 -0.2927637011038513

C -4.2714039827466674 4.5720558212057458 -0.3225816247800957

C -1.8046120760893238 4.5724653813027007 -0.3227630562099737

C 0.6658919536516104 -6.1258468388772958 -0.3285297012081294

C 3.1347911363846981 -6.1280637624268195 -0.3180147534239843

C 5.6064230063698828 -6.1298028029938232 -0.3160904719381961

C -6.7435865986097889 -6.1283240477478147 -0.3185694850741818

C -4.2721196110263993 -6.1259160149798930 -0.3286708531199096

C -1.8026124975109674 -6.1251100673118355 -0.3336639978692855

C 0.6673030884790530 -3.9875339598322013 -0.3223191893181863

C 3.1367120552957486 -3.9903075402874362 -0.3295080289243693

C 5.6059826829924377 -3.9911698176472936 -0.3345456082555565

C -6.7473596330133701 -3.9913086382664713 -0.3349780910474625

C -4.2772129090650957 -3.9905003923548326 -0.3300305380061079

C -1.8045481549434959 -3.9875654507429710 -0.3224571315567708

C 0.6634908325469260 -1.8530737696934345 -0.2787622267545355

C 3.1380997165522055 -1.8540295001510554 -0.2875811057007773

C 5.6074594168642742 -1.8521210121314686 -0.3249233377453038

C -6.7472121680505417 -1.8518378434639695 -0.3374202498900480

C -4.2792646159040100 -1.8525597601924462 -0.3254913584210275

C -1.8119194339291553 -1.8540320943313837 -0.2882705460885440

C 1.4870580518343948 1.7280241635986702 -0.0946959452067713

C 3.9755761617240930 1.7280362686722430 -0.0920015218751207

C 6.4418412884060858 1.7179814490041796 -0.2585720359218584

C -5.9194061159456188 1.7160264472393294 -0.3229186060806661

C -3.4549925385487050 1.7160011365337784 -0.3237428230053556

C -0.9909951626417941 1.7178917206345261 -0.2616106992240557

C 1.4858170655049756 3.8623888098731722 -0.2542719038816408

C 3.9632825684098512 3.8613884542655157 -0.2505271861283358

C 6.4416091620835845 3.8624662643841354 -0.2525800877157796

C -5.9172432255216991 3.8584176846231575 -0.3031879775942237

C -3.4504271713058312 3.8578807876735688 -0.3260985506054261

C -0.9829961489179242 3.8587328337102704 -0.3043911117611273

C 1.4886152230539507 5.9983900281213050 -0.3157769921497771

C 3.9573229065096820 5.9944057357606990 -0.3047257354388826

C 6.4319913378491895 5.9943260556319462 -0.3052719730511519

C -5.9182106842915854 5.9982208418406362 -0.3158882374712242

C -3.4495629702933068 5.9976637912823936 -0.3295953897856752

C -0.9805753958420578 5.9978084068014983 -0.3295598384707237

C 1.4903231695899803 -4.7015743160273473 -0.3287646467331111

C 3.9595158971289086 -4.7037133400421611 -0.3304718703606575

C 6.4284007615306438 -4.7058010040996168 -0.3319359821680621

C -5.9237436971325543 -4.7039634993551740 -0.3310172910977176

C -3.4520781082185295 -4.7017688275141261 -0.3290552050238169

C -0.9803661165359898 -4.7007453842802001 -0.3284836450226738

C 1.4907851984287754 -2.5654543687361988 -0.2982866571666367

C 3.9622779558333088 -2.5658081850850705 -0.3191269627470881

C 6.4299294366543647 -2.5656790850352627 -0.3358951979516860

C -5.9255144552000942 -2.5659345110544218 -0.3364059293512192

C -3.4580142364834652 -2.5661170196716392 -0.3196466976590067

C -0.9862833808565039 -2.5654594683568757 -0.2983636972842447

C 1.4786004947580791 -0.4414182551356820 -0.2029886998485964

C 3.9674823784301365 -0.4327853965200488 -0.2365977600288147

C 6.4322952662793957 -0.4251097963557218 -0.3157427199328353

C -5.9232765710200592 -0.4247739314168095 -0.3345531361976875

C -3.4562675454759009 -0.4253996660931687 -0.3166866860139912

C -1.0002809968010808 -0.4330090768419943 -0.2385956909835834

Pt 2.5965712959314073 1.5109153494052965 4.2271962896372806

Pt 1.1705925925506255 1.2071066219517101 2.0778088130851291

Pt 3.6781944697942093 1.2044424746969256 2.0824753249912114

O 2.3006393464369017 1.0208663760852845 6.0324623291642769

O 1.6004534955213012 -0.1632530569660648 6.5583008186832821

Gr-(CO/Pt_6)

C 0.9763078184796123 0.1440805985504934 -0.3008935991305410

C 1.0232655062963962 2.3235413129819094 -0.1031392288014832

C 0.9837760671250431 4.4436994609819145 -0.3437307699070864

C 0.9791486308348327 -6.2459579621099319 -0.4501553380376819

C 0.9786530027481488 -4.1120375428766280 -0.4838144377006675

C 0.9769308366473295 -1.9793402362045232 -0.4455386017917764

C 3.4490274585912699 0.1624046668825629 -0.4105685220896689

C 3.4767378321299818 2.3092594101881758 -0.3466755016388703

C 3.4508705181544306 4.4436699326434894 -0.4484662670780235

C 3.4459086971905402 -6.2454266154833302 -0.5041425370871373

C 3.4441361462217985 -4.1105479156144966 -0.5017713618213131

C 3.4439128624160249 -1.9753581573282109 -0.4703696741349468

C 5.9109887443360902 0.1643770826325728 -0.4589784511929604

C 5.9206124365668424 2.3016478212500981 -0.4635480271050643

C 5.9195936864900949 4.4394392148202648 -0.4995036638229884

C 5.9151049691723978 -6.2441214912958509 -0.5398875710396034

C 5.9097253520011739 -4.1121190156524925 -0.5208265765258862

C 5.9097091044173800 -1.9743858632811531 -0.4735986326378185

C -6.4329385115922895 0.1616536135408504 -0.3873068380595406

C -6.4231044241199395 2.3021469266912042 -0.4520273405767004

C -6.4187447496050609 4.4383744354705108 -0.4994183490967767

C -6.4199467161974377 -6.2457424784875055 -0.5398528255161956

C -6.4202289271109247 -4.1082540781498675 -0.5195964314577184

C -6.4314738322310516 -1.9802682602786952 -0.4310367055698254

C -3.9728139948778325 0.1433217796032489 -0.1637599167908519

C -3.9724529753213043 2.3109232964446669 -0.2802919686855301

C -3.9549889390106379 4.4371916461251288 -0.4292505575929102

C -3.9534255688365620 -6.2477319500597543 -0.5078312732762047

C -3.9545648749890825 -4.1132450296418241 -0.5169670701555367

C -3.9542969516288737 -1.9799116366478522 -0.4351293628568538

C -1.4992369681991580 0.1376460234875188 -0.2714433157432588

C -1.4795886138349088 2.3193663402510172 -0.0130188680107572

C -1.4835186374408558 4.4396513018268919 -0.3178278585817154

C -1.4876587393869842 -6.2475970863809662 -0.4596187137531373

C -1.4882889098265020 -4.1139752053689032 -0.4933332169092974

C -1.4886110573044462 -1.9802757428878071 -0.4403540076180743

C 1.8271298374479956 1.5807215343785774 -0.1809526325787836

C 1.8207217094088604 3.7456709679801143 -0.3254389107524904

C 1.8031786772382494 5.8638138768862298 -0.4468136213256528

C 1.8011287658417006 -4.8220330411564838 -0.4855977384563825

C 1.8002961707699727 -2.6886697778741357 -0.4727469036391927

C 1.7993424637031223 -0.5552216776287882 -0.3887361612922735

C 4.2804599237859753 1.5884836172761734 -0.4101082169209960

C 4.2779038233620570 3.7280483346609401 -0.4346243891561006

C 4.2707574252699425 5.8640148095774176 -0.5147590796785702

C 4.2664895790037365 -4.8223525757062955 -0.5188052639330083

C 4.2649192616128966 -2.6861336099747022 -0.4885520686099500

C 4.2661813097633106 -0.5483432886234745 -0.4519577611841470

C 6.7356820320523783 1.5877685240658694 -0.4657812553488831

C 6.7401694628576925 3.7264941523820330 -0.4906875557680159

C 6.7386098824739848 5.8625314365072132 -0.5355208380344640

C 6.7346153707224952 -4.8216145923734359 -0.5338146728067619

C 6.7292457920291939 -2.6882362679871576 -0.4832700389058662

C 6.7288845194622118 -0.5500694169826561 -0.4486743213357105

C -5.6077417314450164 1.5927914212555869 -0.3875550152408014

C -5.5986119717769753 3.7262911187383541 -0.4623576028517657

C -5.5980298929439964 5.8614538737235211 -0.5204891302429537

C -5.5976500931226045 -4.8214236169312841 -0.5336455866294925

C -5.6011768764418433 -2.6886283106456355 -0.4644164508550066

C -5.6267402385362351 -0.5627900058435031 -0.3324861026016084

C -3.1676872726355643 1.5892367171683883 -0.0757360606055268

C -3.1376238740620477 3.7271843652752548 -0.3354999404337189

C -3.1320656029417275 5.8614398736961331 -0.4671104159862196

C -3.1317730015672538 -4.8242471312843360 -0.5128236063336606

C -3.1316497852820206 -2.6901261363203863 -0.4750860849329257

C -3.1262303957127622 -0.5658775999273535 -0.3179410578904847

C -0.6731612235092435 1.5715969243861663 -0.0501250485515179

C -0.6545580835291371 3.7458042708680832 -0.2223473463011238

C -0.6640743874319579 5.8614971243079186 -0.4147365214474732

C -0.6658445464706677 -4.8247903606971665 -0.4846265423689591

C -0.6654930594730368 -2.6906896523648736 -0.4640663104155003

C -0.6661233471290631 -0.5600096468061651 -0.3518245868081031

Pt -3.9011780991736016 0.1547060110001652 4.4157032207668419

Pt -0.8940718243923946 1.5925947801844682 4.5588628563538158

Pt 1.9188046296917607 2.8315058260867261 4.3404242728373443

Pt -1.0170409346553226 1.7788538709960982 2.0270417434936530

Pt 1.7008689634757941 2.1218126541411557 1.9638657719867432

Pt -3.9488222156820219 0.6317631229250953 1.9539759113516801

C -2.5153333354883918 1.9616766465595177 5.0811724105771194

O -2.6612340848759315 2.8474022574779490 5.5323420346515118

Gr-(NO/Pt_6)

C 0.9663617299871072 0.1453740099353134 -0.3210299052182215

C 1.0130113368927915 2.3264355199867834 -0.1352254886776691

C 0.9768648939469582 4.4468005697400441 -0.3635741003420794

C 0.9708113095468054 -6.2438952306210727 -0.4624537408037384

C 0.9700588607170442 -4.1100484216180133 -0.4923858494949283

C 0.9678438631221002 -1.9771523524739205 -0.4561334181994692

C 3.4410337594891054 0.1644188084275813 -0.4288848375921717

C 3.4678796730193522 2.3119740538099007 -0.3667536991254163

C 3.4443186282721108 4.4467217228414411 -0.4628212619222616

C 3.4393255312363316 -6.2422804888435337 -0.5140020081519250

C 3.4358193467933589 -4.1080109051253313 -0.5135035926465292

C 3.4350639815944533 -1.9738814189863048 -0.4857357302384901

C 5.9033471872529475 0.1659670481188114 -0.4762210030845875

C 5.9128460260545115 2.3030044889409540 -0.4776520778269777

C 5.9126361253894544 4.4419135778864449 -0.5115289582263802

C 5.9082810396177994 -6.2407428636668438 -0.5471705253852548

C 5.9025889192986618 -4.1090896446977290 -0.5309311341748773

C 5.9018746397856301 -1.9724256515299938 -0.4917887814401212

C -6.4395552721393052 0.1618240598103817 -0.4105449029852668

C -6.4292221466679340 2.3034213347925645 -0.4629096051520101

C -6.4266718943304255 4.4403549675097755 -0.5070583154423485

C -6.4276810902497683 -6.2435698776060180 -0.5431155960215532

C -6.4271061412545452 -4.1051897280855716 -0.5203959167990364

C -6.4379170080845531 -1.9753025154077495 -0.4444922487054583

C -3.9809197687502005 0.1474762327358574 -0.1692705952905325

C -3.9771645762025467 2.3119741498712036 -0.3035813269557828

C -3.9631447170767613 4.4386934128895019 -0.4399813192573347

C -3.9613530338267844 -6.2456260972294091 -0.5107214598764918

C -3.9629172143419811 -4.1106472320660137 -0.5134849406757862

C -3.9630496752069604 -1.9778161273095600 -0.4284832900414308

C -1.5086038590470423 0.1408642354150871 -0.2755951869866546

C -1.4892685941556572 2.3195422676587478 -0.0751913207642865

C -1.4917776136441088 4.4411336340840384 -0.3412474401497914

C -1.4961659572164683 -6.2455145624016044 -0.4679206493302761

C -1.4972190977908300 -4.1124007615092015 -0.4953885529050321

C -1.4974260336571543 -1.9784488424988922 -0.4407706519317465

C 1.8165591988980960 1.5837112670820659 -0.2062555675227635

C 1.8131869777013794 3.7477229139315122 -0.3445315820423236

C 1.7954398136518446 5.8658190623267705 -0.4598551118278884

C 1.7927705779108063 -4.8199418021860874 -0.4960497473558441

C 1.7916738069507261 -2.6870032439994120 -0.4840816715837608

C 1.7904024623383332 -0.5532332518851346 -0.4066937423223198

C 4.2724581029110542 1.5901138621437472 -0.4276424465169768

C 4.2701698449670662 3.7304797302591490 -0.4513424713918681

C 4.2639763368001891 5.8670947054351545 -0.5245051082719598

C 4.2594633815188185 -4.8197903346365250 -0.5286147941322339

C 4.2560904812169955 -2.6845323122704579 -0.5037545697749835

C 4.2582375600624189 -0.5465268359318705 -0.4687796011151057

C 6.7281461293577447 1.5888510076697866 -0.4799097383419380

C 6.7324907103587979 3.7283675602450037 -0.5017864283891544

C 6.7316334928681361 5.8648040307782301 -0.5426017137641654

C 6.7273046033793644 -4.8189922631915829 -0.5402406657898062

C 6.7220683055844752 -2.6850520698395282 -0.4983079677028925

C 6.7220939883847262 -0.5489785943800589 -0.4693713286282115

C -5.6140029820076007 1.5936091387482483 -0.4016417591714223

C -5.6056761992236366 3.7277221930042481 -0.4714075598758320

C -5.6062303254374868 5.8630077461544188 -0.5246748668894634

C -5.6053394842268407 -4.8191422123038272 -0.5323717800927632

C -5.6082668966917524 -2.6863408877580821 -0.4638592039701628

C -5.6329316275973653 -0.5599131775985571 -0.3553399770655421

C -3.1645833241604318 1.5940086103731970 -0.1229621889798302

C -3.1445981124592817 3.7285623543405260 -0.3556057661063168

C -3.1402077245265461 5.8630040752338060 -0.4740013005869326

C -3.1398324845246659 -4.8225835047247578 -0.5124506303854028

C -3.1406176861949322 -2.6884532270772366 -0.4702601776551845

C -3.1364091725440759 -0.5631977431444977 -0.3144344844610210

C -0.6815128036952860 1.5766659277388779 -0.0744477955802836

C -0.6631449069147530 3.7453007288941231 -0.2551429344873792

C -0.6719211803514105 5.8632489079876011 -0.4286893240783023

C -0.6747798617098519 -4.8232317481427422 -0.4904898830445141

C -0.6743690823341675 -2.6886601080462253 -0.4678415335884019

C -0.6751622079582216 -0.5583763344664989 -0.3586420987007752

Pt -3.8341936022823169 0.3613137655809640 4.4378783491915801

Pt -0.8764833814165733 1.5761018755320446 4.5510324798786534

Pt 1.9342357524671758 2.5825903002409527 4.3649773318459530

Pt -1.0015137086833779 1.7417160738604744 2.0453674007345288

Pt 1.7050501163212672 2.0894157198915648 1.9445169033983856

Pt -3.9142218911428781 0.6979891660262885 1.9807590722939814

N -2.3930210991871856 1.8489093003056611 5.5825063631803804

O -2.3772350567520002 2.8679682550209291 5.8894990524920292

Gr-(N_2/Pt_6)

C 1.0242104596622248 0.1909614987177033 -0.3790228841439660

C 1.0620142436320830 2.3681342931173512 -0.2076730172537840

C 1.0351713190889633 4.4933269632984940 -0.3891681412273744

C 1.0326544537521354 -6.1959119549300841 -0.4695686086130557

C 1.0319619523442558 -4.0623418610449997 -0.5064219659911533

C 1.0289856856595492 -1.9304702755309302 -0.4906385240109650

C 3.5007082242842831 0.2112660270780244 -0.4461018122828744

C 3.5242585399736583 2.3573839299890880 -0.3810253694548837

C 3.5044831798122988 4.4935687276743268 -0.4679697385601251

C 3.5005266779659117 -6.1946864080949036 -0.5076947808635310

C 3.4984698838248587 -4.0602657200079406 -0.5112667568818097

C 3.4973881700493816 -1.9257601877281729 -0.4946663212687596

C 5.9645364190710275 0.2132235948903336 -0.4811742230729052

C 5.9717513316718005 2.3497689974057758 -0.4782981280332841

C 5.9714680872491464 4.4877045928560593 -0.5055998837450799

C 5.9679647416891228 -6.1953788202234907 -0.5404575955718824

C 5.9647142523835743 -4.0620259235996370 -0.5293356473668709

C 5.9646403965043362 -1.9243429536254073 -0.4947380366237777

C -6.3757678262240818 0.2128378326617240 -0.4447377800977463

C -6.3694910595559859 2.3504998420919039 -0.4835615016894348

C -6.3669908638419352 4.4857638329734018 -0.5093307972577321

C -6.3680084317076151 -6.1983874269104691 -0.5452473943487925

C -6.3682874058516923 -4.0613332170744805 -0.5447928791292274

C -6.3749262270251856 -1.9298717222896578 -0.4851251692983016

C -3.9090280921584033 0.2032205956257886 -0.3572724916063343

C -3.9127447809072904 2.3545401244448341 -0.3876911370544747

C -3.9014647312662563 4.4843444840744917 -0.4628396457590949

C -3.9008497087400826 -6.1997280187549366 -0.5218322987420967

C -3.9028533937929852 -4.0649003619530477 -0.5463551081326550

C -3.9034095443006334 -1.9311764619615222 -0.5134194019793279

C -1.4460423064071311 0.1899097957119409 -0.3846002311042671

C -1.4358786768695726 2.3572725627517732 -0.1696127097259588

C -1.4313276868330664 4.4873632730991089 -0.3792505155157393

C -1.4346874469772697 -6.1985702988559472 -0.4842924878187880

C -1.4362059454538150 -4.0658193763016719 -0.5233224356560342

C -1.4373884100702847 -1.9327592470114405 -0.5062105654769171

C 1.8689398014246930 1.6278189496940056 -0.2536743361560418

C 1.8702994871962488 3.7928952750927136 -0.3743233563184276

C 1.8567778625044156 5.9140436535466820 -0.4642753736181859

C 1.8553174934274654 -4.7717893776818645 -0.5001074789169442

C 1.8535107266774862 -2.6392751396211604 -0.5024384285113435

C 1.8505239854465678 -0.5067773718044508 -0.4430718413947119

C 4.3305582857404854 1.6367025315456489 -0.4352218729200938

C 4.3291378270509249 3.7772651552544589 -0.4537250046225836

C 4.3244727625428609 5.9137690890926455 -0.5172166118737822

C 4.3213690394976059 -4.7722070908840148 -0.5213825765422397

C 4.3194639468796971 -2.6361378135970885 -0.5045859999536875

C 4.3193030108664683 -0.4991939991891856 -0.4776772455041680

C 6.7885193905938745 1.6363045951521409 -0.4859180461435493

C 6.7917425286393023 3.7742231878648242 -0.4989905628013620

C 6.7907883759720491 5.9106414930785212 -0.5372030468656677

C 6.7879109870822205 -4.7729823717110094 -0.5428450296246226

C 6.7854503910351074 -2.6378688888897703 -0.5066302342790161

C 6.7841960286514347 -0.5006574408864175 -0.4767515996456773

C -5.5523344862427555 1.6401177890932592 -0.4514156819380837

C -5.5458862697222822 3.7737612383146244 -0.4869553478890616

C -5.5463012654981849 5.9087207698633284 -0.5279271080580719

C -5.5460967878168805 -4.7740270293735874 -0.5515641589173441

C -5.5482408572538091 -2.6399282201216399 -0.5193595217018609

C -5.5607643593448861 -0.5071610445124684 -0.4255265562703308

C -3.1054277604453011 1.6330074439633160 -0.2701331462946950

C -3.0829782467596454 3.7738786727430549 -0.4030018331155460

C -3.0788973440230807 5.9093361813571432 -0.4890063095475465

C -3.0795494648780339 -4.7764966447471799 -0.5349051344437266

C -3.0809720424707230 -2.6423313707919984 -0.5316389464168596

C -3.0789574545666234 -0.5131523345877858 -0.4426925288445034

C -0.6280294977715348 1.6187453199899950 -0.1912994391586178

C -0.6045406065249974 3.7894345337211517 -0.3046685259316817

C -0.6106388031464789 5.9104605667963801 -0.4463672687605271

C -0.6130046311762891 -4.7757289281914765 -0.5086481127204108

C -0.6136680882930765 -2.6420853070439438 -0.5109964573801840

C -0.6152890062842147 -0.5121846343931029 -0.4391791941985304

Pt -3.8525333058088167 0.6962399406496322 4.4829966212364880

Pt -1.0194513834901757 1.5638678307955853 4.4303229432494788

Pt 1.7393322301575562 2.3526015857811702 4.3624068092089896

Pt -1.0507803435613754 1.7051715039187405 1.9809952854664257

Pt 1.6858863254715502 2.1059084093515770 1.8984233332182683

Pt -3.9399182238507371 0.6041143264755977 2.0104149991156870

N -4.4966096246979692 0.6914942817850669 7.4914940857183741

N -4.1731861138652260 0.7020999505436452 6.4045858254249453

Gr-(O_2/Pt_6)

C 1.0210222109512275 0.1909034817187623 -0.3475058997219467

C 1.0670969354102331 2.3704377218411592 -0.1752997231263222

C 1.0325223868014595 4.4935809549674159 -0.3397796527092325

C 1.0281337156614496 -6.1971935978022055 -0.4238787273484466

C 1.0272922698497977 -4.0632361794767053 -0.4629690933335926

C 1.0242105599087692 -1.9310783537847724 -0.4522367760443764

C 3.4972468906499907 0.2114370873678615 -0.4255417865882087

C 3.5223869050539358 2.3588096476556606 -0.3648780714336883

C 3.5026066614455695 4.4943469912605218 -0.4482474261402949

C 3.4964038572922869 -6.1949474258164372 -0.4787441644280062

C 3.4927209081590318 -4.0612413995951098 -0.4738822582551840

C 3.4923322948772073 -1.9269041052742848 -0.4605575184004316

C 5.9598075803615123 0.2133243038876991 -0.4448963088210274

C 5.9683887303074750 2.3497440576191435 -0.4533585420644659

C 5.9697008784209560 4.4896743263630237 -0.4834518165313195

C 5.9647343615493087 -6.1941316747146216 -0.5076602883124348

C 5.9590376308085675 -4.0631579447501780 -0.4852583656946035

C 5.9590092841156386 -1.9255642268239406 -0.4483972125067375

C -6.3830006031701485 0.2092262134606617 -0.3664911256558661

C -6.3736782043294884 2.3515468304961660 -0.4267502813706940

C -6.3710646856195972 4.4873601157682854 -0.4700556887922556

C -6.3711779394139478 -6.1971157610644489 -0.5028179727046851

C -6.3719239995009902 -4.0598943970154391 -0.4815014127084662

C -6.3828817642224598 -1.9312252489853448 -0.4049150484323292

C -3.9226941056673037 0.1908657641339220 -0.1515303450736241

C -3.9239994862664620 2.3617218609392241 -0.2532965905028508

C -3.9072242717115730 4.4859336656695623 -0.3970255704854999

C -3.9049983557700583 -6.1989433985112106 -0.4733787133487350

C -3.9074417363323306 -4.0652795155028247 -0.4875889470958708

C -3.9079961514606154 -1.9329663063689324 -0.4161721480648755

C -1.4520643466856473 0.1868136401437210 -0.2907825756993194

C -1.4244631919091271 2.3660904021826648 -0.0254351768401815

C -1.4339222269128440 4.4885629922574166 -0.2955869068015424

C -1.4397564796193940 -6.1988107626829647 -0.4288990228566085

C -1.4406919978279289 -4.0665828291170403 -0.4729384258771088

C -1.4421625481655020 -1.9335129404371862 -0.4443827786673360

C 1.8692339255992616 1.6293739693250968 -0.2376418764613302

C 1.8686352723303044 3.7920083639996927 -0.3415248168999678

C 1.8523267829837335 5.9130681404903394 -0.4267789687831680

C 1.8501316226593501 -4.7729038501293246 -0.4592223895841681

C 1.8487497266298165 -2.6406775419629911 -0.4647736456320430

C 1.8459092284131122 -0.5073246890195966 -0.4158897583569416

C 4.3281833210820642 1.6363018760462600 -0.4176508739600351

C 4.3268157316569544 3.7790805439673205 -0.4377721161042061

C 4.3212345609387244 5.9144235179571814 -0.4928785677393854

C 4.3159403144417672 -4.7734929668091812 -0.4868214318137873

C 4.3131743077055491 -2.6375125758763547 -0.4661162108594787

C 4.3152854409195536 -0.4992637397626192 -0.4494544766239237

C 6.7840080929620070 1.6361797404674414 -0.4494779541494633

C 6.7890319673955188 3.7756010107548343 -0.4718059680943334

C 6.7881211064454812 5.9117750532782170 -0.5052546747800788

C 6.7829897428297476 -4.7728233885821396 -0.4957848467704498

C 6.7782097690646888 -2.6395040683324993 -0.4522773212115503

C 6.7788739867433021 -0.5019791468718102 -0.4272545359278848

C -5.5575578199886948 1.6425364380595093 -0.3611627301742821

C -5.5498491780214207 3.7748328618486342 -0.4325059066773207

C -5.5502617414978266 5.9096973863824500 -0.4854314719082495

C -5.5496661704669918 -4.7733040795780148 -0.4957924216778054

C -5.5538670356175146 -2.6414938578170171 -0.4336144786040368

C -5.5768489048079033 -0.5144423328657242 -0.3178938656754351

C -3.1143393072818166 1.6379109164147598 -0.0489996907747088

C -3.0895716621766538 3.7756875496478957 -0.3082861730806528

C -3.0835329919939101 5.9102882332696440 -0.4339033357049598

C -3.0836621887338400 -4.7767276615085912 -0.4837240070877744

C -3.0857013892439982 -2.6434830785094547 -0.4599684080684430

C -3.0812916723095958 -0.5188002645605359 -0.3116020858735808

C -0.6222425200423070 1.6219853323045923 -0.1045880916869848

C -0.6033211797682645 3.7941219107421444 -0.2176319475426514

C -0.6152192758366253 5.9093339446318742 -0.3870241279862636

C -0.6180596834634811 -4.7769032367133546 -0.4595600490799505

C -0.6178922801725308 -2.6429380609743012 -0.4617319022279212

C -0.6201848970614936 -0.5134197662462556 -0.3799023979498326

Pt -4.0723090798303572 0.5242029000638926 4.4876482295889488

Pt -0.9075174372868231 1.8591076502882453 4.5258821410009240

Pt 1.6755436447013414 2.1632481279058551 4.3960532787982967

Pt -0.9442297706661096 1.7885473464529467 2.0194915841031840

Pt 1.7348829783928243 2.0687812266853038 1.9285391962485186

Pt -3.8699540099251841 0.7650480692114581 1.9545159389624427

O -5.9245091294091798 -0.9284551930062008 4.4974787345497909

O -2.4992041653316868 2.1837133989213493 5.1381867847193643
